# A structural homology approach to identify potential cross-reactive antibody responses following SARS-CoV-2 infection

**DOI:** 10.1038/s41598-022-15225-3

**Published:** 2022-07-06

**Authors:** Joseph R. McGill, H. A. Daniel Lagassé, Nancy Hernandez, Louis Hopkins, Wojciech Jankowski, Quinn McCormick, Vijaya Simhadri, Basil Golding, Zuben E. Sauna

**Affiliations:** Division of Plasma Protein Therapeutics, Office of Tissues and Advanced Therapies, Center for Biologics Evaluation and Research, Food and Drug Administration, Silver Spring, MD USA

**Keywords:** Computational biology and bioinformatics, Immunology, Molecular modelling

## Abstract

The emergence of the novel SARS-CoV-2 virus is the most important public-health issue of our time. Understanding the diverse clinical presentations of the ensuing disease, COVID-19, remains a critical unmet need. Here we present a comprehensive listing of the diverse clinical indications associated with COVID-19. We explore the theory that anti-SARS-CoV-2 antibodies could cross-react with endogenous human proteins driving some of the pathologies associated with COVID-19. We describe a novel computational approach to estimate structural homology between SARS-CoV-2 proteins and human proteins. Antibodies are more likely to interrogate 3D-structural epitopes than continuous linear epitopes. This computational workflow identified 346 human proteins containing a domain with high structural homology to a SARS-CoV-2 Wuhan strain protein. Of these, 102 proteins exhibit functions that could contribute to COVID-19 clinical pathologies. We present a testable hypothesis to delineate unexplained clinical observations vis-à-vis COVID-19 and a tool to evaluate the safety-risk profile of potential COVID-19 therapies.

## Introduction

In late 2019, cases of severe pneumonia with unknown etiology were reported in Wuhan, China. The novel coronavirus, SARS-CoV-2, was identified as the causative agent for this disease, called coronavirus disease 2019 (COVID-19). COVID-19 was declared a pandemic in March 2020^1^; according to the World Health Organization (WHO) dashboard (https://covid19.who.int/), as of June 8, 2022, there have been 530,896,347 confirmed cases and 6,301,020 deaths due to COVID-19 globally.

COVID-19 has three consecutive stages of increasing severity^2^. In the early stage, flu-like symptoms appear followed by viral pneumonia. The second stage is characterized by pulmonary inflammation and coagulopathy with increased levels of inflammatory biomarkers. The third stage of the disease is associated with fibrosis. Disease severity and mortality is associated with higher levels of inflammatory markers and increased serum levels of inflammatory cytokines^3^. Moreover, numerous clinical symptoms and pathologies have been reported in individual COVID-19 patients. Data also suggests that the causative virus, SARS-CoV-2, could act as a trigger for the development of a rapid autoimmune responses^4–6^. For example, Guillain-Barré syndrome, an immune-mediated disorder where the cross-reactivity of anti-pathogen antibodies with host proteins plays an important role, has been associated with COVID-19^7,8^.

Following an infection, the physiological role of the immune system is to identify and eliminate the pathogen. However, pathogenic infections have also been associated with autoimmunity wherein aberrant immune responses are elicited against host proteins. Such immune responses may be linked to numerous human diseases, e.g., diabetes mellitus type 1, systemic lupus erythematosus, celiac disease, Henoch-Schönlein purpura, sarcoidosis, Graves’ disease and idiopathic thrombocytopenic purpura^9,10^. One mechanism that may contribute to autoimmunity involves pathogen-derived antigens that are similar to host antigens but differ enough to induce an immune response^11^.

Several computational studies^12,13^ have sought to identify homologous regions between pathogen-derived proteins and human proteins. However, these methods, which are based on sequence homology, cannot capture structural homologies. Here, we present a novel strategy for comparing the surface structure of individual chains between two different proteins.

A critical mass of biomedical information associating COVID-19 infections with autoimmune diseases has emerged^14–16^ [for reviews see^17–19^]. Additionally, a study^20^ has reported the result of a high-throughput assay to detect autoantibodies in 194 SARS-CoV-2 infected COVID-19 patients. The study found an increase in autoantibodies in the COVID-19 patients compared to uninfected controls. This report provides a comprehensive survey of clinical pathologies associated with SARS-CoV-2 infection and the human proteins that could be associated with these pathologies. Additionally, we describe a novel computational tool to compare the 3D structures of SARS-CoV-2 proteins and human proteins. We list 102 human genes with high structural homology to SARS-CoV-2 proteins. We do not claim that these 102 human genes are necessarily linked to human disease. These data sets are “hypothesis generating” and constitute a useful resource for scientists and clinicians.

## Materials and methods

### Obtaining PDB files

PDB files were obtained from the Research Collaboratory for Structural Bioinformatics Protein Data Bank (RCSB PDB)^21^, an open access repository of protein structural information. The data set analyzed 22,867 protein structure files. These PDB files were split into two subsets of files, 22,556 human protein files and 26 SARS-CoV-2 protein files. These structures are not all unique proteins; the complete list of all PDB accession codes for human and SARS-CoV-2 proteins used in this study is provided in Supplementary Tables 1 and 2 respectively. The proteins were split into macromolecular chains to prepare the data for input into the ProBiS^22^ algorithm.

### Generating surface areas

For each protein/chain, solvent accessible surface atoms are calculated^23^. The surface calculation identifies vertices based on physiochemical properties and is done individually for each protein chain within the ProBiS algorithm. These surface calculations are pre-computed and locally stored to speed up the later computations.

### Aligning proteins

The ProBiS algorithm searches for structurally similar sites on a local scale by finding all matches to each query protein chain within the proteome list supplied. The alignment of two protein chains, one from the query protein and one from the human proteome, is based on finding similar regions between the two chains.

The benefit of the ProBiS algorithm to this exercise is that it is focused on local alignments. The algorithm will begin with vertices relating to three amino acid residue surface regions and expand outwards along the backbone.

### Drawing protein structures

Graphical protein structure were created using PyMol v 2.3.5^24^.

### Quantification and statistical analysis

Each match between a query chain and a proteome chain is scored in four ways:

- *Surface vectors angle* A vector orthogonal to the geometric mean of the surface of each protein at the aligned area is calculated. The angle between these two vectors is calculated. If the angle is less than 90° (1.571 rad), the alignment will be retained in the results.
- *Surface patch root mean squared distance (RMSD)* The distance between each set of vertices in the match is calculated and the RMSD is calculated from this from the following formula.
  $${\text{Distance}} = \sqrt {\left( {x_{1} - x_{2} } \right)^{2} + \left( {y_{1} - y_{2} } \right)^{2} + \left( {z_{1} - z_{2} } \right)^{2} }$$
  $${\text{RMSD}} = \sqrt {\frac{1}{n }\mathop \sum \limits_{i = 1}^{n} \left( {Distance_{i} } \right)^{2} }$$
  where: Each vertex exists in three-dimensional space and has x, y, and z coordinates. There are *n* sets of vertices.
- *Surface patch size* The algorithm requires that alignments must contain at least 10 vertices.
- *E-values* E-values for a particular alignment are calculate using the Karlin-Altschul Equation^25^ in a similar fashion to evaluating the quality of matches in a sequence alignment according to the Karlin-Altschul Equation:
  $$E=kmn{e}^{-lS}$$
  where: *E* = The Expect-value of the alignment (E-value); *k* and *λ* are constants (k = 0.134 and λ = 0.3176, these are the often-used values for an ungapped alignment in a structural homology search); *m* is the number of vertices in the query (SARS-CoV-2) alignment fragment; *n* is the number of vertices in the library (human protein); *S* is the substitution score calculated using the sum of scored for each substitution using a BLOSUM62 matrix.

### Go Term and KEGG pathway analysis

Both analyses were performed in R^26^ and calculated using the topGO^27^ package. The universe of genes for both analyses were the list of proteins for which a PDB file was available rather than the entire human proteome. All p-values were adjusted using the Benjamini–Hochberg method^28^ as implemented in R. A threshold of 0.05 was used as a cut off for significance using adjusted p-values.

### Data visualizations

All Figures were created using ggplot2^29^.

### Data and Code availability

A list of PDB IDs and chain IDs for both human and SARS-CoV-2 proteins has been provided in the supplementary data along with all code used to generate the alignments (Supplementary Tables 1 and 2 and Supplementary File-Codes-1). The URLs for the tools and databases used in this study are provided in Supplementary Table 3.

## Results

### The overall approach and selection of structures for determining cross-reactivity of anti-SARS-CoV-2 antibodies with endogenous human proteins

**Figure 1:**
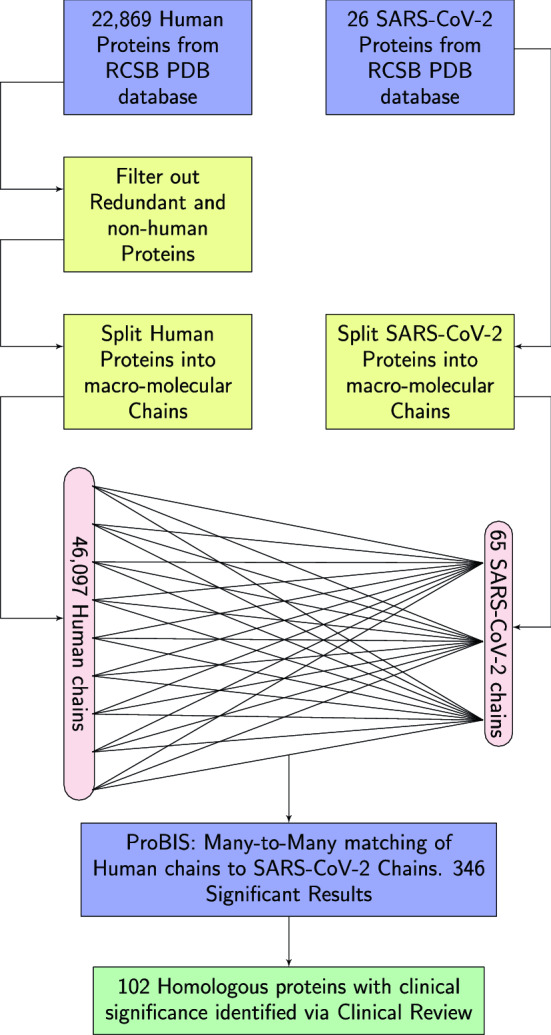
Structural homology identification workflow. A description of our workflow for identifying the steps for identifying homologous protein structures. First, obtain PDB files human and SARS-CoV-2 Wuhan strain proteins. Second, filter out redundant and non-endogenous human proteins. Third, split all proteins into macro-molecular chains. Fourth, find matches between SARS-CoV-2 chains and human chains. Fifth, identify which chains found in step 4, belong to proteins which could have clinical indications related to COVID-19.

The overarching goal of this theoretical study is to identify endogenous human proteins that could plausibly be targeted by antibodies that are elicited against a pathogen. Previous computational strategies have used sequence homology to identify human proteins that may be similar to viral antigens^12,13,30,31^. Here we have adopted a novel strategy that seeks to identify structural homologies using SARS-CoV-2 as a model system. The workflow of our strategy to identify conformational homology between SARS-CoV-2 proteins and endogenous human proteins is illustrated in Fig. 1. An important limitation of the method we adopted is the available protein structures represent approximately 35% of the human proteome, despite large increases in publicly available datasets in recent years. However, researchers tend to solve the structures of proteins deemed biologically or clinically important. Thus, the available human protein structures are likely to represent those most likely associated with clinical indications.

### Identification of human proteins with structural homology to SARS-CoV-2 proteins

A detailed description of the novel computational workflow we used to identify endogenous human proteins with structural homology to SARS-CoV-2 proteins has been provided in the “Methods” section. The human and SARS-CoV-2 proteins used in this analysis are listed in the Supplementary Tables 1 and 2 respectively. Identifying structural homologies relies on parsing databases of known protein structural files down to non-redundant lists of host organism proteins, splitting those proteins into distinct macro-molecular chains, generating surface structures for each chain, and matching endogenous human chains to SARS-CoV-2 protein chains.

**Figure 2:**
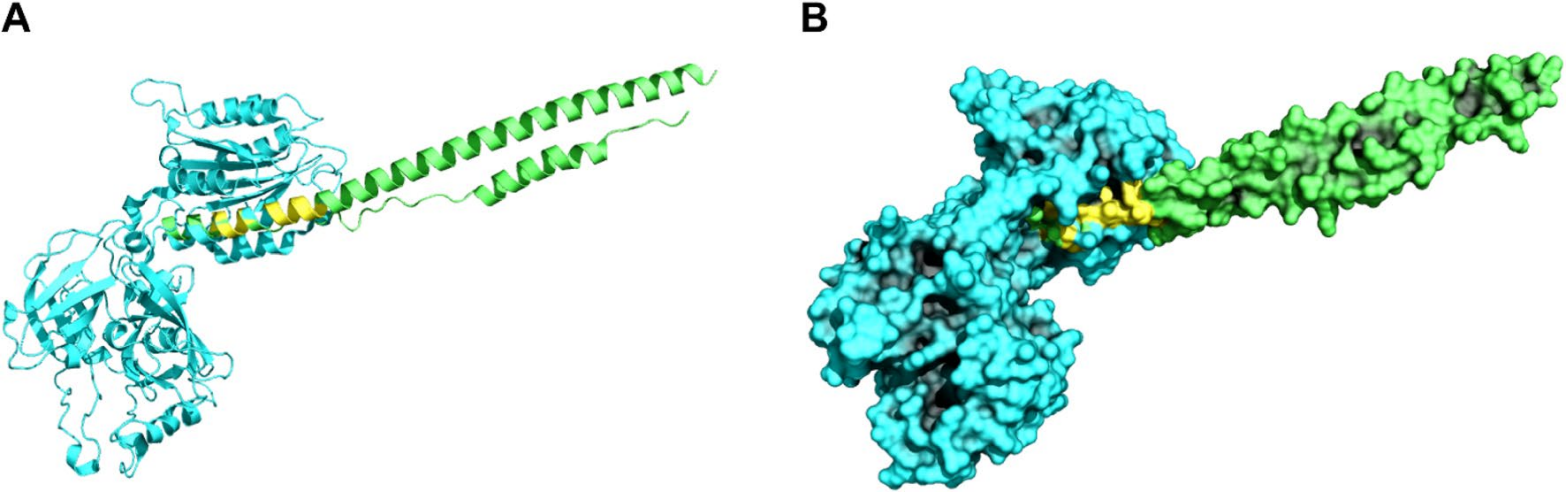
Surface matches between SARS-CoV-2 and complement factor B. We have included both a cartoon diagram **(A)** and a surface diagram **(B)** to show one of the human proteins (Complement Factor B, PDB ID: 1RTK, chain A, cyan) with structural homology to a SARS-CoV-2 protein (Spike Protein S2 subunit, PDB ID: 6LXT, chain A, green). This match (yellow) satisfied all of our criteria for inclusion as a significant match with an RMSD of 0.2 Å, an alignment score of 6.7, 15 aligned vertices, and sva value of 0.3 and an e-value of 1.43 × 10^–5^.

For this study, the criterion for homology between a SARS-CoV-2 and an endogenous human protein was based on four criteria. Proteins were considered structurally homologous based on: (i) the angles of the orthogonal vectors of the surface patches, (ii) the root mean squared distances between each set of vertices, (iii) the size of the surface patches, and (iv) the e-value of the match. The rationale for these criteria was built into the software implementation of the ProBiS algorithm^22,23^. Using these criteria, we identified 346 human proteins with structural homology to SARS-CoV-2 proteins. These proteins are listed in Supplementary Table 4. A full record of the matches including alignment lengths, chains and scores is provided in Supplemental Table 5. An illustrative example is provided in Fig. 2, showing the structural alignment between SARS-CoV-2 spike protein chain A (Protein Data Bank (PDB) ID: 6LXT) and the human protein complement factor B (PDB ID: 1RTK).

### Clinical indications associated with SARS-CoV-2 infections

A list of COVID-19 -related clinical pathologies was compiled following a review of the medical literature. COVID-19 related clinical pathologies were categorized as general (involving multiple organ systems) or the immune system (immunopathology), or specific to an individual organ system including lungs (pneumopathy), heart (cardiopathy), blood and clotting (hemopathy and coagulopathy), liver (hepatopathy), kidney (nephropathy), gastrointestinal, or brain (neuropathy). In Table 1 we provide a comprehensive list of the clinical indications and symptoms associated with SARS-CoV-2 infections and provide a numerical code for each indication/symptom. The numerical codes were used for easy cross-referencing to human proteins that could potentially be (i) associated with COVID-19 related clinical pathologies based on their known function and (ii) targeted by anti-SARS-CoV-2 antibodies (see below).

**Table 1:** Clinical indications and symptoms associated with SARS-CoV-2 infections.

Reference code	Clinical indication
1.0	General
1.1	Fever
1.2	Cytotoxic injury
1.3	Multiple organ failure
1.4	Systemic vasculitis
1.5	Necrosis
1.6	Edema
1.7	Purpuric skin rash
1.8	Myalgia (muscle pain)
1.9	Fatigue
1.10	Myopathy
1.11	Refractory metabolic acidosis
2.0	Lungs/ Pneumopathy
2.1	Pneumonia (Atypical, severe, acute)
2.2	Respiratory epithelium disruption
2.3	Lung (pulmonary, respiratory) failure
2.4	Pulmonary defects
2.5	Hyaline membrane formation (alveolar walls)
2.6	Epithelial cell proliferation
2.7	Epithelial Cell desquamation
2.8	Increased fibrosis
2.9	Inflammation(pneumonitis)
2.10	Cough
2.11	Dyspnea
2.12	Pulmonary consolidation
2.13	Alveolar damage
2.14	Edema
2.15	Pneumocyte hyperplasia
2.16	Hypoxemia
2.17	Acute Respiratory Distress Syndrome (ARDS)
2.18	Pulmonary embolism
3.0	Heart/Cardiopathy
3.1	Cardiovascular complications
3.2	Heart Damage
3.3	Myocarditis
3.4	Cardiovascular injury
3.5	Pericarditis
3.6	Arrhythmia
3.7	Cardiac arrest
3.8	Cardiomyopathy
3.9	Heart failure
3.10	Cardiogenic shock
3.11	Hypotension
3.12	Tachycardia
3.13	Bradycardia
3.14	Reversible cardiomegaly
3.15	Transient atrial fibrillation
4.0	Blood and clotting, hemopathy, & coagulopathy
4.1	Thrombocytopenia
4.2	Hemorrhage
4.3	Disseminated intravascular coagulation (DIC)
4.4	Venous thromboembolism
4.5	Coagulopathy
4.6	Sepsis-induced coagulopathy
4.7	Sepsis/septic Shock
4.8	Thrombosis
4.9	Microvascular injury
4.10	Hyperferritinemi
4.11	Kawasaki Shock Syndrome (KSS)
4.12	Venous thromboembolism (VTE)
4.13	Decreased albumin
4.14	Increased C-reactive protein
4.15	Increased LDH
4.16	Increased erythrocyte sedimentation rate (ESR)
5.0	Immune system Immunopathology
5.1	Immune cell imbalance
5.2	Cytokine dysregulation
5.3	Chemokine dysregulation
5.4	Cytokine Storm
5.5	Cytokine release syndrome
5.6	Multinucleated epithelial giant cell formation
5.7	Immune thrombocytopenic purpura
5.8	Immune cell infiltration
5.9	Complement-mediated cytotoxicity
5.10	Complement deposition
5.11	Pediatric COVID-19 Hyperinflammatory Syndrome (PeCOHS)
5.12	Pediatric inflammatory multisystem syndrome (PIMS)
5.13	NK cell deficiency
5.14	Lymphopenia
6.0	Liver/hepatopathy
6.1	Mild hepatitis
6.2	Liver Damage
6.3	Hepatic dysfunction
7.0	Kidney/Nephropathy
7.1	Kidney damage
7.2	Renal impairment
8.0	Gastrointestinal
8.1	Gastrointestinal complications
8.2	Diarrhea
8.3	Nausea
8.4	Vomiting
9.0	Brain/neuropathy
9.1	Guillain–Barre Syndrome (GBS)
9.2	Miller Fisher Syndrome (MFS)
9.3	Polyneuritis cranialis
9.4	Ischemic Stroke
9.5	Mental confusion
9.6	Anosmia (loss of smell)
9.7	Ageusia (loss of taste)
9.8	Intracerebral hemorrhage
9.9	Altered consciousness
9.10	Dizziness
9.11	Cerebral venous sinus thrombosis
9.12	Encephalitis
9.13	Seizures
9.14	Refractory status epilepticus
9.15	Acute disseminated encephalomyelitis (ADEM)
9.16	Leukoencephalopathy
9.17	Headache

### Human structural homologues of SARS-CoV-2 proteins and clinical pathologies associated with COVID-19

We also assessed which of the 346 human proteins found to have structural homology to SARS-CoV-2 could be associated with reported COVID-19 pathologies. General literature search strategies included the gene name in addition to key terms such as coronavirus, molecular mimicry, autoimmunity, pathology, knock-out/down in some combination within the PubMed search engine. In some instances, no hits were available to provide evidence of connection to COVID-19 pathologies. However, theoretical associations were hypothesized based on protein localization and function. Protein candidates were tiered. Proteins expressed on the plasma membrane surface or secreted into the extracellular space were considered to have the highest probability of being bound and inhibited by a cross-reactive anti-SARS-CoV-2 antibody. Based on this strategy, of the 346 human proteins that are structural homologs of SARS-CoV-2 proteins, 102 were identified as having biological functions that could be associated with COVID-19 pathologies or symptoms upon inhibition by cross-reactive antibody responses. We have tabulated and encoded clinical indications associated with COVID-19. The COVID-19-related clinical indications potentially associated with each of these 102 human proteins are shown in Table 2.

**Table 2:** Human proteins with both structural homology to SARS-CoV-2 proteins and associations with clinical indications.

Gene symbol	Entrez Id	Protein name	Protein description	Reference codes
AAK1	22,848	AP2-associated protein kinase 1	Regulates clathrin-mediated endocytosis by phosphorylating the AP2M1/mu2 subunit of the adaptor protein complex 2 (AP-2) which ensures high affinity binding of AP-2 to cargo membrane proteins during the initial stages of endocytosis. Isoform 1 and isoform 2 display similar levels of kinase activity towards AP2M1. Preferentially, may phosphorylate substrates on threonine residues. Regulates phosphorylation of other AP-2 subunits as well as AP-2 localization and AP-2-mediated internalization of ligand complexes. Phosphorylates NUMB and regulates its cellular localization, promoting NUMB localization to endosomes. Binds to and stabilizes the activated form of NOTCH1, increases its localization in endosomes and regulates its transcriptional activity	9
ACE	1636	Angiotensin-converting enzyme	Converts angiotensin I to angiotensin II by release of the terminal His-Leu, this results in an increase of the vasoconstrictor activity of angiotensin. Also able to inactivate bradykinin, a potent vasodilator. Has also a glycosidase activity which releases GPI-anchored proteins from the membrane by cleaving the mannose linkage in the GPI moiety	2.0, 2.4, 2.17
ACVR2A	92	Activin receptor type-2A	On ligand binding, forms a receptor complex consisting of two type II and two type I transmembrane serine/threonine kinases. Type II receptors phosphorylate and activate type I receptors which autophosphorylate, then bind and activate SMAD transcriptional regulators. Receptor for activin A, activin B and inhibin A. Mediates induction of adipogenesis by GDF6 (By similarity)	1.0, 1.6
ADORA2A	135	Adenosine receptor A2	Receptor for adenosine. The activity of this receptor is mediated by G proteins which activate adenylyl cyclase	2.0, 2.3, 2.9
ADSL	158	Adenylosuccinate lyase	Catalyzes two non-sequential steps in de novo AMP synthesis: converts (S)-2-(5-amino-1-(5-phospho-D-ribosyl)imidazole-4-carboxamido)succinate (SAICAR) to fumarate plus 5-amino-1-(5-phospho-D-ribosyl)imidazole-4-carboxamide, and thereby also contributes to de novo IMP synthesis, and converts succinyladenosine monophosphate (SAMP) to AMP and fumarate	9.0, 9.13
AGXT	189	Serine–pyruvate aminotransferase		7.0, 7.1, 7.2
AHCYL2	23,382	Adenosylhomocysteinase 3	May regulate the electrogenic sodium/bicarbonate cotransporter SLC4A4 activity and Mg(2 +)-sensitivity. On the contrary of its homolog AHCYL1, does not regulate ITPR1 sensitivity to inositol 1,4,5-trisphosphate	1.0, 1.6, 3.0, 3.10
ALB	213	Albumin	Binds water, Ca(2 +), Na( +), K( +), fatty acids, hormones, bilirubin and drugs (Probable). Its main function is the regulation of the colloidal osmotic pressure of blood (Probable). Major zinc transporter in plasma, typically binds about 80% of all plasma zinc. Major calcium and magnesium transporter in plasma, binds approximately 45% of circulating calcium and magnesium in plasma (By similarity). Potentially has more than two calcium-binding sites and might additionally bind calcium in a non-specific manner (By similarity). The shared binding site between zinc and calcium at residue Asp-273 suggests a crosstalk between zinc and calcium transport in the blood (By similarity). The rank order of affinity is zinc > calcium > magnesium (By similarity). Binds to the bacterial siderophore enterobactin and inhibits enterobactin-mediated iron uptake of E.coli from ferric transferrin and may thereby limit the utilization of iron and growth of enteric bacteria such as E.coli. Does not prevent iron uptake by the bacterial siderophore aerobactin	1.0, 1.6, 1.9
ALDH1A1	216	Retinal dehydrogenase 1	Can convert/oxidize retinaldehyde to retinoic acid. Binds free retinal and cellular retinol-binding protein-bound retinal (By similarity). May have a broader specificity and oxidize other aldehydes in vivo.", NA)	4.0, 4.1, 2.4
ALOX5AP	241	Arachidonate 5-lipoxygenase-activating protein	Required for leukotriene biosynthesis by ALOX5 (5-lipoxygenase). Anchors ALOX5 to the membrane. Binds arachidonic acid and could play an essential role in the transfer of arachidonic acid to ALOX5. Binds to MK-886, a compound that blocks the biosynthesis of leukotrienes	1.0, 1.6, 3.0, 3.10
AOC1	26	Amiloride-sensitive amine oxidase	Catalyzes the degradation of compounds such as putrescine, histamine, spermine, and spermidine, substances involved in allergic and immune responses, cell proliferation, tissue differentiation, tumor formation, and possibly apoptosis. Placental DAO is thought to play a role in the regulation of the female reproductive function	8.0, 8.2, 9.0, 9.17
APP	351	ABPP	N-APP binds TNFRSF21 triggering caspase activation and degeneration of both neuronal cell bodies (via caspase-3) and axons (via caspase-6)	3.0, 3.1, 9.0, 9.13
ARHGEF1	9138	Rho guanine nucleotide exchange factor 1, isoform CRA_e	Seems to play a role in the regulation of RhoA GTPase by guanine nucleotide-binding alpha-12 (GNA12) and alpha-13 (GNA13) subunits. Acts as GTPase-activating protein (GAP) for GNA12 and GNA13, and as guanine nucleotide exchange factor (GEF) for RhoA GTPase. Activated G alpha 13/GNA13 stimulates the RhoGEF activity through interaction with the RGS-like domain. This GEF activity is inhibited by binding to activated GNA12. Mediates angiotensin-2-induced RhoA activation	2.0, 5.0, 5.7
AURKA	6790	Aurora kinase A	Mitotic serine/threonine kinase that contributes to the regulation of cell cycle progression. Associates with the centrosome and the spindle microtubules during mitosis and plays a critical role in various mitotic events including the establishment of mitotic spindle, centrosome duplication, centrosome separation as well as maturation, chromosomal alignment, spindle assembly checkpoint, and cytokinesis. Required for normal spindle positioning during mitosis and for the localization of NUMA1 and DCTN1 to the cell cortex during metaphase. Required for initial activation of CDK1 at centrosomes. Phosphorylates numerous target proteins, including ARHGEF2, BORA, BRCA1, CDC25B, DLGP5, HDAC6, KIF2A, LATS2, NDEL1, PARD3, PPP1R2, PLK1, RASSF1, TACC3, p53/TP53 and TPX2. Regulates KIF2A tubulin depolymerase activity. Required for normal axon formation. Plays a role in microtubule remodeling during neurite extension. Important for microtubule formation and/or stabilization. Also acts as a key regulatory component of the p53/TP53 pathway, and particularly the checkpoint-response pathways critical for oncogenic transformation of cells, by phosphorylating and stabilizing p53/TP53. Phosphorylates its own inhibitors, the protein phosphatase type 1 (PP1) isoforms, to inhibit their activity. Necessary for proper cilia disassembly prior to mitosis. Regulates protein levels of the anti-apoptosis protein BIRC5 by suppressing the expression of the SCF(FBXL7) E3 ubiquitin-protein ligase substrate adapter FBXL7 through the phosphorylation of the transcription factor FOXP1	8.0, 8.2, 8.3, 8.4
BCR	613	Breakpoint cluster region protein	Protein with a unique structure having two opposing regulatory activities toward small GTP-binding proteins. The C-terminus is a GTPase-activating protein (GAP) domain which stimulates GTP hydrolysis by RAC1, RAC2 and CDC42. Accelerates the intrinsic rate of GTP hydrolysis of RAC1 or CDC42, leading to down-regulation of the active GTP-bound form. The central Dbl homology (DH) domain functions as guanine nucleotide exchange factor (GEF) that modulates the GTPases CDC42, RHOA and RAC1. Promotes the conversion of CDC42, RHOA and RAC1 from the GDP-bound to the GTP-bound form. The amino terminus contains an intrinsic kinase activity. Functions as an important negative regulator of neuronal RAC1 activity (By similarity). Regulates macrophage functions such as CSF1-directed motility and phagocytosis through the modulation of RAC1 activity. Plays a major role as a RHOA GEF in keratinocytes being involved in focal adhesion formation and keratinocyte differentiation	1.0, 1.6
BICC1	80,114	Protein bicaudal C homolog 1	Putative RNA-binding protein. Acts as a negative regulator of Wnt signaling. May be involved in regulating gene expression during embryonic development	7.0, 7.2, 9.0, 9.17
BMPR2	659	Bone morphogenetic protein receptor type-2	On ligand binding, forms a receptor complex consisting of two type II and two type I transmembrane serine/threonine kinases. Type II receptors phosphorylate and activate type I receptors which autophosphorylate, then bind and activate SMAD transcriptional regulators. Binds to BMP7, BMP2 and, less efficiently, BMP4. Binding is weak but enhanced by the presence of type I receptors for BMPs. Mediates induction of adipogenesis by GDF6	2.0, 2.11
BTK	695	Tyrosine-protein kinase BTK	Non-receptor tyrosine kinase indispensable for B lymphocyte development, differentiation and signaling. Binding of antigen to the B-cell antigen receptor (BCR) triggers signaling that ultimately leads to B-cell activation. After BCR engagement and activation at the plasma membrane, phosphorylates PLCG2 at several sites, igniting the downstream signaling pathway through calcium mobilization, followed by activation of the protein kinase C (PKC) family members. PLCG2 phosphorylation is performed in close cooperation with the adapter protein B-cell linker protein BLNK. BTK acts as a platform to bring together a diverse array of signaling proteins and is implicated in cytokine receptor signaling pathways. Plays an important role in the function of immune cells of innate as well as adaptive immunity, as a component of the Toll-like receptors (TLR) pathway. The TLR pathway acts as a primary surveillance system for the detection of pathogens and are crucial to the activation of host defense. Especially, is a critical molecule in regulating TLR9 activation in splenic B-cells. Within the TLR pathway, induces tyrosine phosphorylation of TIRAP which leads to TIRAP degradation. BTK plays also a critical role in transcription regulation. Induces the activity of NF-kappa-B, which is involved in regulating the expression of hundreds of genes. BTK is involved on the signaling pathway linking TLR8 and TLR9 to NF-kappa-B. Transiently phosphorylates transcription factor GTF2I on tyrosine residues in response to BCR. GTF2I then translocates to the nucleus to bind regulatory enhancer elements to modulate gene expression. ARID3A and NFAT are other transcriptional target of BTK. BTK is required for the formation of functional ARID3A DNA-binding complexes. There is however no evidence that BTK itself binds directly to DNA. BTK has a dual role in the regulation of apoptosis	8.0, 8.2
C1S	716	Complement C1s subcomponent	C1s B chain is a serine protease that combines with C1q and C1r to form C1, the first component of the classical pathway of the complement system. C1r activates C1s so that it can, in turn, activate C2 and C4	5.0, 5.10
CD27	939	CD27 antigen	Receptor for CD70/CD27L. May play a role in survival of activated T-cells. May play a role in apoptosis through association with SIVA1	5.0, 5.1, 5.2, 5.3, 5.14
CFB	629	C3/C5 convertase	Factor B which is part of the alternate pathway of the complement system is cleaved by factor D into 2 fragments: Ba and Bb. Bb, a serine protease, then combines with complement factor 3b to generate the C3 or C5 convertase. It has also been implicated in proliferation and differentiation of preactivated B-lymphocytes, rapid spreading of peripheral blood monocytes, stimulation of lymphocyte blastogenesis and lysis of erythrocytes. Ba inhibits the proliferation of preactivated B-lymphocytes	1.0, 1.1, 1.6, 1.9, 2.11, 4.0, 4.5, 4.9, 4.13, 5.0, 7.0, 7.1, 7.2
CLCN1	1180	Chloride channel protein 1	Voltage-gated chloride channel. Plays an important role in membrane repolarization in skeletal muscle cells after muscle contraction. The CLC channel family contains both chloride channels and proton-coupled anion transporters that exchange chloride or another anion for protons (Probable). The absence of conserved gating glutamate residues is typical for family members that function as channels (Probable)	1.0, 1.8, 1.10
CNTF	1270	Ciliary neurotrophic factor	CNTF is a survival factor for various neuronal cell types. Seems to prevent the degeneration of motor axons after axotomy	9.0, 9.1
COMT	1312	Catechol O-methyltransferase	Catalyzes the O-methylation, and thereby the inactivation, of catecholamine neurotransmitters and catechol hormones	9.0, 9.9, 9.10, 9.13
CRP	1401	C-reactive protein	Displays several functions associated with host defense: it promotes agglutination, bacterial capsular swelling, phagocytosis and complement fixation through its calcium-dependent binding to phosphorylcholine. Can interact with DNA and histones and may scavenge nuclear material released from damaged circulating cells	1.0, 1.2, 1.4, 3.0, 3.1, 3.2, 3.4, 3.9, 4.0, 4.5, 4.7, 4.9, 5.0, 5.1, 5.2, 5.9, 5.10, 5.14, 7.0, 7.1, 7.2, 9.0, 9.5, 9.13, 9.17
CTNNB1	1499	Catenin , beta 1, 88 kDa, isoform CRA_a	Key downstream component of the canonical Wnt signaling pathway. In the absence of Wnt, forms a complex with AXIN1, AXIN2, APC, CSNK1A1 and GSK3B that promotes phosphorylation on N-terminal Ser and Thr residues and ubiquitination of CTNNB1 via BTRC and its subsequent degradation by the proteasome. In the presence of Wnt ligand, CTNNB1 is not ubiquitinated and accumulates in the nucleus, where it acts as a coactivator for transcription factors of the TCF/LEF family, leading to activate Wnt responsive genes. Involved in the regulation of cell adhesion, as component of an E-cadherin:catenin adhesion complex (By similarity). Acts as a negative regulator of centrosome cohesion. Involved in the CDK2/PTPN6/CTNNB1/CEACAM1 pathway of insulin internalization. Blocks anoikis of malignant kidney and intestinal epithelial cells and promotes their anchorage-independent growth by down-regulating DAPK2. Disrupts PML function and PML-NB formation by inhibiting RANBP2-mediated sumoylation of PML. Promotes neurogenesis by maintaining sympathetic neuroblasts within the cell cycle (By similarity). Involved in chondrocyte differentiation via interaction with SOX9: SOX9-binding competes with the binding sites of TCF/LEF within CTNNB1, thereby inhibiting the Wnt signaling (By similarity)	1.0, 1.10, 2.0, 2.11, 2.16, 3.0, 3.2, 3.6, 3.7, 3.11, 4.0, 4.5, 4.9, 5.0, 6.0, 6.2
CTSS	1520	Cathepsin S	Thiol protease. Key protease responsible for the removal of the invariant chain from MHC class II molecules and MHC class II antigen presentation. The bond-specificity of this proteinase is in part similar to the specificities of cathepsin L	1.0, 1.9, 2.0, 2.2, 2.8, 2.10, 3.0, 3.2, 7.0, 7.1, 7.2
CXCL12	6387	Stromal cell-derived factor 1	Chemoattractant active on T-lymphocytes and monocytes but not neutrophils. Activates the C-X-C chemokine receptor CXCR4 to induce a rapid and transient rise in the level of intracellular calcium ions and chemotaxis. SDF-1-beta(3–72) and SDF-1-alpha(3–67) show a reduced chemotactic activity. Binding to cell surface proteoglycans seems to inhibit formation of SDF-1-alpha(3–67) and thus to preserve activity on local sites. Also binds to atypical chemokine receptor ACKR3, which activates the beta-arrestin pathway and acts as a scavenger receptor for SDF-1. Binds to the allosteric site (site 2) of integrins and activates integrins ITGAV:ITGB3, ITGA4:ITGB1 and ITGA5:ITGB1 in a CXCR4-independent manner. Acts as a positive regulator of monocyte migration and a negative regulator of monocyte adhesion via the LYN kinase. Stimulates migration of monocytes and T-lymphocytes through its receptors, CXCR4 and ACKR3, and decreases monocyte adherence to surfaces coated with ICAM-1, a ligand for beta-2 integrins. SDF1A/CXCR4 signaling axis inhibits beta-2 integrin LFA-1 mediated adhesion of monocytes to ICAM-1 through LYN kinase. Inhibits CXCR4-mediated infection by T-cell line-adapted HIV-1. Plays a protective role after myocardial infarction. Induces down-regulation and internalization of ACKR3 expressed in various cells. Has several critical functions during embryonic development; required for B-cell lymphopoiesis, myelopoiesis in bone marrow and heart ventricular septum formation. Stimulates the proliferation of bone marrow-derived B-cell progenitors in the presence of IL7 as well as growth of stromal cell-dependent pre-B-cells (By similarity)	1.0, 1.8, 1.9, 3.0, 3.11, 5.0, 5.1, 5.3, 5.14, 8.0, 8.2, 8.3, 8.4, 9.0, 9.10, 9.17
DPP8	54,878	Dipeptidyl-peptidase 8, isoform CRA_f	Dipeptidyl peptidase that cleaves off N-terminal dipeptides from proteins having a Pro or Ala residue at position 2	1.0, 1.8, 1.10, 5.0, 5.2, 5.3, 5.5, 5.11, 9.0
EPHA2	1969	Receptor protein-tyrosine kinase	Receptor tyrosine kinase which binds promiscuously membrane-bound ephrin-A family ligands residing on adjacent cells, leading to contact-dependent bidirectional signaling into neighboring cells. The signaling pathway downstream of the receptor is referred to as forward signaling while the signaling pathway downstream of the ephrin ligand is referred to as reverse signaling. Activated by the ligand ephrin-A1/EFNA1 regulates migration, integrin-mediated adhesion, proliferation and differentiation of cells. Regulates cell adhesion and differentiation through DSG1/desmoglein-1 and inhibition of the ERK1/ERK2 (MAPK3/MAPK1, respectively) signaling pathway. May also participate in UV radiation-induced apoptosis and have a ligand-independent stimulatory effect on chemotactic cell migration. During development, may function in distinctive aspects of pattern formation and subsequently in development of several fetal tissues. Involved for instance in angiogenesis, in early hindbrain development and epithelial proliferation and branching morphogenesis during mammary gland development. Engaged by the ligand ephrin-A5/EFNA5 may regulate lens fiber cells shape and interactions and be important for lens transparency development and maintenance. With ephrin-A2/EFNA2 may play a role in bone remodeling through regulation of osteoclastogenesis and osteoblastogenesis	5.0, 5.4
EPHA8	2046	Ephrin type-A receptor 8	Receptor tyrosine kinase which binds promiscuously GPI-anchored ephrin-A family ligands residing on adjacent cells, leading to contact-dependent bidirectional signaling into neighboring cells. The signaling pathway downstream of the receptor is referred to as forward signaling while the signaling pathway downstream of the ephrin ligand is referred to as reverse signaling. The GPI-anchored ephrin-A EFNA2, EFNA3, and EFNA5 are able to activate EPHA8 through phosphorylation. With EFNA5 may regulate integrin-mediated cell adhesion and migration on fibronectin substrate but also neurite outgrowth. During development of the nervous system plays also a role in axon guidance. Downstream effectors of the EPHA8 signaling pathway include FYN which promotes cell adhesion upon activation by EPHA8 and the MAP kinases in the stimulation of neurite outgrowth (By similarity)	5.0, 9.0
EPHB3	2049	Ephrin type-B receptor 3	Receptor tyrosine kinase which binds promiscuously transmembrane ephrin-B family ligands residing on adjacent cells, leading to contact-dependent bidirectional signaling into neighboring cells. The signaling pathway downstream of the receptor is referred to as forward signaling while the signaling pathway downstream of the ephrin ligand is referred to as reverse signaling. Generally has an overlapping and redundant function with EPHB2. Like EPHB2, functions in axon guidance during development regulating for instance the neurons forming the corpus callosum and the anterior commissure, 2 major interhemispheric connections between the temporal lobes of the cerebral cortex. In addition to its role in axon guidance plays also an important redundant role with other ephrin-B receptors in development and maturation of dendritic spines and the formation of excitatory synapses. Controls other aspects of development through regulation of cell migration and positioning. This includes angiogenesis, palate development and thymic epithelium development for instance. Forward and reverse signaling through the EFNB2/EPHB3 complex also regulate migration and adhesion of cells that tubularize the urethra and septate the cloaca. Finally, plays an important role in intestinal epithelium differentiation segregating progenitor from differentiated cells in the crypt	1.0, 1.5
EZR	7430	Ezrin	Probably involved in connections of major cytoskeletal structures to the plasma membrane. In epithelial cells, required for the formation of microvilli and membrane ruffles on the apical pole. Along with PLEKHG6, required for normal macropinocytosis	1.0, 9.0
FCGR2B	2213	Low affinity immunoglobulin gamma Fc region receptor II-b	Receptor for the Fc region of complexed or aggregated immunoglobulins gamma. Low affinity receptor. Involved in a variety of effector and regulatory functions such as phagocytosis of immune complexes and modulation of antibody production by B-cells. Binding to this receptor results in down-modulation of previous state of cell activation triggered via antigen receptors on B-cells (BCR), T-cells (TCR) or via another Fc receptor. Isoform IIB1 fails to mediate endocytosis or phagocytosis. Isoform IIB2 does not trigger phagocytosis	5.0, 5.14
FCGR3A	2214	Low affinity immunoglobulin gamma Fc region receptor III-A	Receptor for the Fc region of IgG. Binds complexed or aggregated IgG and also monomeric IgG. Mediates antibody-dependent cellular cytotoxicity (ADCC) and other antibody-dependent responses, such as phagocytosis	5.0, 5.14
FGFR1	2260	Fibroblast growth factor receptor 1	Tyrosine-protein kinase that acts as cell-surface receptor for fibroblast growth factors and plays an essential role in the regulation of embryonic development, cell proliferation, differentiation and migration. Required for normal mesoderm patterning and correct axial organization during embryonic development, normal skeletogenesis and normal development of the gonadotropin-releasing hormone (GnRH) neuronal system. Phosphorylates PLCG1, FRS2, GAB1 and SHB. Ligand binding leads to the activation of several signaling cascades. Activation of PLCG1 leads to the production of the cellular signaling molecules diacylglycerol and inositol 1,4,5-trisphosphate. Phosphorylation of FRS2 triggers recruitment of GRB2, GAB1, PIK3R1 and SOS1, and mediates activation of RAS, MAPK1/ERK2, MAPK3/ERK1 and the MAP kinase signaling pathway, as well as of the AKT1 signaling pathway. Promotes phosphorylation of SHC1, STAT1 and PTPN11/SHP2. In the nucleus, enhances RPS6KA1 and CREB1 activity and contributes to the regulation of transcription. FGFR1 signaling is down-regulated by IL17RD/SEF, and by FGFR1 ubiquitination, internalization and degradation	1
FGFR3	2261	Fibroblast growth factor receptor 3	Tyrosine-protein kinase that acts as cell-surface receptor for fibroblast growth factors and plays an essential role in the regulation of cell proliferation, differentiation and apoptosis. Plays an essential role in the regulation of chondrocyte differentiation, proliferation and apoptosis, and is required for normal skeleton development. Regulates both osteogenesis and postnatal bone mineralization by osteoblasts. Promotes apoptosis in chondrocytes, but can also promote cancer cell proliferation. Required for normal development of the inner ear. Phosphorylates PLCG1, CBL and FRS2. Ligand binding leads to the activation of several signaling cascades. Activation of PLCG1 leads to the production of the cellular signaling molecules diacylglycerol and inositol 1,4,5-trisphosphate. Phosphorylation of FRS2 triggers recruitment of GRB2, GAB1, PIK3R1 and SOS1, and mediates activation of RAS, MAPK1/ERK2, MAPK3/ERK1 and the MAP kinase signaling pathway, as well as of the AKT1 signaling pathway. Plays a role in the regulation of vitamin D metabolism. Mutations that lead to constitutive kinase activation or impair normal FGFR3 maturation, internalization and degradation lead to aberrant signaling. Over-expressed or constitutively activated FGFR3 promotes activation of PTPN11/SHP2, STAT1, STAT5A and STAT5B. Secreted isoform 3 retains its capacity to bind FGF1 and FGF2 and hence may interfere with FGF signaling	7.0, 7.1, 7.2, 9.0, 9.1
FGFR4	2264	Fibroblast growth factor receptor	Tyrosine-protein kinase that acts as cell-surface receptor for fibroblast growth factors and plays a role in the regulation of cell proliferation, differentiation and migration, and in regulation of lipid metabolism, bile acid biosynthesis, glucose uptake, vitamin D metabolism and phosphate homeostasis. Required for normal down-regulation of the expression of CYP7A1, the rate-limiting enzyme in bile acid synthesis, in response to FGF19. Phosphorylates PLCG1 and FRS2. Ligand binding leads to the activation of several signaling cascades. Activation of PLCG1 leads to the production of the cellular signaling molecules diacylglycerol and inositol 1,4,5-trisphosphate. Phosphorylation of FRS2 triggers recruitment of GRB2, GAB1, PIK3R1 and SOS1, and mediates activation of RAS, MAPK1/ERK2, MAPK3/ERK1 and the MAP kinase signaling pathway, as well as of the AKT1 signaling pathway. Promotes SRC-dependent phosphorylation of the matrix protease MMP14 and its lysosomal degradation. FGFR4 signaling is down-regulated by receptor internalization and degradation; MMP14 promotes internalization and degradation of FGFR4. Mutations that lead to constitutive kinase activation or impair normal FGFR4 inactivation lead to aberrant signaling	1
FLT3	2322	Receptor-type tyrosine-protein kinase FLT3	Tyrosine-protein kinase that acts as cell-surface receptor for the cytokine FLT3LG and regulates differentiation, proliferation and survival of hematopoietic progenitor cells and of dendritic cells. Promotes phosphorylation of SHC1 and AKT1, and activation of the downstream effector MTOR. Promotes activation of RAS signaling and phosphorylation of downstream kinases, including MAPK1/ERK2 and/or MAPK3/ERK1. Promotes phosphorylation of FES, FER, PTPN6/SHP, PTPN11/SHP-2, PLCG1, and STAT5A and/or STAT5B. Activation of wild-type FLT3 causes only marginal activation of STAT5A or STAT5B. Mutations that cause constitutive kinase activity promote cell proliferation and resistance to apoptosis via the activation of multiple signaling pathways	1.0, 5.0
FOLH1	2346	Glutamate carboxypeptidase 2	Also exhibits a dipeptidyl-peptidase IV type activity. In vitro, cleaves Gly-Pro-AMC	1.0, 8.0, 9.0
FTH1	2495	Ferritin	Stores iron in a soluble, non-toxic, readily available form. Important for iron homeostasis. Iron is taken up in the ferrous form and deposited as ferric hydroxides after oxidation	4.0, 6.0
FTL	2512	Ferritin	Stores iron in a soluble, non-toxic, readily available form. Important for iron homeostasis. Iron is taken up in the ferrous form and deposited as ferric hydroxides after oxidation	4.0, 6.0
GAA	2548	Lysosomal alpha-glucosidase	Essential for the degradation of glycogen in lysosomes. Has highest activity on alpha-1,4-linked glycosidic linkages, but can also hydrolyze alpha-1,6-linked glucans	1.0, 1.8, 1.9, 1.10
GAD1	2571	Glutamate decarboxylase 1 isoform 1	Catalyzes the production of GABA	1.0, 4.0
GBP1	2633	Guanylate-binding protein 1	Hydrolyzes GTP to GMP in 2 consecutive cleavage reactions. Exhibits antiviral activity against influenza virus. Promotes oxidative killing and delivers antimicrobial peptides to autophagolysosomes, providing broad host protection against different pathogen classes	5.0, 5.1, 5.2
GEM	2669	GTP-binding protein	Could be a regulatory protein, possibly participating in receptor-mediated signal transduction at the plasma membrane. Has guanine nucleotide-binding activity but undetectable intrinsic GTPase activity	1.0, 2.0, 7.0
GPC1	2817	Glypican-1	Cell surface proteoglycan that bears heparan sulfate	1.0, 6.0
GRIA2	2891	Glutamate receptor 2	Receptor for glutamate that functions as ligand-gated ion channel in the central nervous system and plays an important role in excitatory synaptic transmission. L-glutamate acts as an excitatory neurotransmitter at many synapses in the central nervous system. Binding of the excitatory neurotransmitter L-glutamate induces a conformation change, leading to the opening of the cation channel, and thereby converts the chemical signal to an electrical impulse. The receptor then desensitizes rapidly and enters a transient inactive state, characterized by the presence of bound agonist. In the presence of CACNG4 or CACNG7 or CACNG8, shows resensitization which is characterized by a delayed accumulation of current flux upon continued application of glutamate. Through complex formation with NSG1, GRIP1 and STX12 controls the intracellular fate of AMPAR and the endosomal sorting of the GRIA2 subunit toward recycling and membrane targeting (By similarity)	9
GRID2	2895	Glutamate receptor ionotropic, delta-2	Receptor for glutamate. L-glutamate acts as an excitatory neurotransmitter at many synapses in the central nervous system. The postsynaptic actions of Glu are mediated by a variety of receptors that are named according to their selective agonists. Promotes synaptogenesis and mediates the D-Serine-dependent long term depression signals and AMPA receptor endocytosis of cerebellar parallel fiber-Purkinje cell (PF-PC) synapses through the beta-NRX1-CBLN1-GRID2 triad complex	9
GSK3B	2932	Glycogen synthase kinase-3 beta	Constitutively active protein kinase that acts as a negative regulator in the hormonal control of glucose homeostasis, Wnt signaling and regulation of transcription factors and microtubules, by phosphorylating and inactivating glycogen synthase (GYS1 or GYS2), EIF2B, CTNNB1/beta-catenin, APC, AXIN1, DPYSL2/CRMP2, JUN, NFATC1/NFATC, MAPT/TAU and MACF1. Requires primed phosphorylation of the majority of its substrates. In skeletal muscle, contributes to insulin regulation of glycogen synthesis by phosphorylating and inhibiting GYS1 activity and hence glycogen synthesis. May also mediate the development of insulin resistance by regulating activation of transcription factors. Regulates protein synthesis by controlling the activity of initiation factor 2B (EIF2BE/EIF2B5) in the same manner as glycogen synthase. In Wnt signaling, GSK3B forms a multimeric complex with APC, AXIN1 and CTNNB1/beta-catenin and phosphorylates the N-terminus of CTNNB1 leading to its degradation mediated by ubiquitin/proteasomes. Phosphorylates JUN at sites proximal to its DNA-binding domain, thereby reducing its affinity for DNA. Phosphorylates NFATC1/NFATC on conserved serine residues promoting NFATC1/NFATC nuclear export, shutting off NFATC1/NFATC gene regulation, and thereby opposing the action of calcineurin. Phosphorylates MAPT/TAU on 'Thr-548', decreasing significantly MAPT/TAU ability to bind and stabilize microtubules. MAPT/TAU is the principal component of neurofibrillary tangles in Alzheimer disease. Plays an important role in ERBB2-dependent stabilization of microtubules at the cell cortex. Phosphorylates MACF1, inhibiting its binding to microtubules which is critical for its role in bulge stem cell migration and skin wound repair. Probably regulates NF-kappa-B (NFKB1) at the transcriptional level and is required for the NF-kappa-B-mediated anti-apoptotic response to TNF-alpha (TNF/TNFA). Negatively regulates replication in pancreatic beta-cells, resulting in apoptosis, loss of beta-cells and diabetes. Through phosphorylation of the anti-apoptotic protein MCL1, may control cell apoptosis in response to growth factors deprivation. Phosphorylates MUC1 in breast cancer cells, decreasing the interaction of MUC1 with CTNNB1/beta-catenin. Is necessary for the establishment of neuronal polarity and axon outgrowth. Phosphorylates MARK2, leading to inhibit its activity. Phosphorylates SIK1 at 'Thr-182', leading to sustain its activity. Phosphorylates ZC3HAV1 which enhances its antiviral activity. Phosphorylates SNAI1, leading to its BTRC-triggered ubiquitination and proteasomal degradation. Phosphorylates SFPQ at 'Thr-687' upon T-cell activation. Phosphorylates NR1D1 st 'Ser-55' and 'Ser-59' and stabilizes it by protecting it from proteasomal degradation. Regulates the circadian clock via phosphorylation of the major clock components including ARNTL/BMAL1, CLOCK and PER2. Phosphorylates CLOCK AT 'Ser-427' and targets it for proteasomal degradation. Phosphorylates ARNTL/BMAL1 at 'Ser-17' and 'Ser-21' and primes it for ubiquitination and proteasomal degradation. Phosphorylates OGT at 'Ser-3' or 'Ser-4' which positively regulates its activity. Phosphorylates MYCN in neuroblastoma cells which may promote its degradation. Regulates the circadian rhythmicity of hippocampal long-term potentiation and ARNTL/BMLA1 and PER2 expression (By similarity). Acts as a regulator of autophagy by mediating phosphorylation of KAT5/TIP60 under starvation conditions, leading to activate KAT5/TIP60 acetyltransferase activity and promote acetylation of key autophagy regulators, such as ULK1 and RUBCNL/Pacer. Negatively regulates extrinsic apoptotic signaling pathway via death domain receptors. Promotes the formation of an anti-apoptotic complex, made of DDX3X, BRIC2 and GSK3B, at death receptors, including TNFRSF10B. The anti-apoptotic function is most effective with weak apoptotic signals and can be overcome by stronger stimulation.", NA)	1.0, 2.0, 7.0, 9.0
GYPA	2993	Glycophorin	Glycophorin A is the major intrinsic membrane protein of the erythrocyte. The N-terminal glycosylated segment, which lies outside the erythrocyte membrane, has MN blood group receptors. Appears to be important for the function of SLC4A1 and is required for high activity of SLC4A1. May be involved in translocation of SLC4A1 to the plasma membrane. Is a receptor for influenza virus. Is a receptor for Plasmodium falciparum erythrocyte-binding antigen 175 (EBA-175); binding of EBA-175 is dependent on sialic acid residues of the O-linked glycans. Appears to be a receptor for Hepatitis A virus (HAV)	4
HCK	3055	Tyrosine-protein kinase	Non-receptor tyrosine-protein kinase found in hematopoietic cells that transmits signals from cell surface receptors and plays an important role in the regulation of innate immune responses, including neutrophil, monocyte, macrophage and mast cell functions, phagocytosis, cell survival and proliferation, cell adhesion and migration. Acts downstream of receptors that bind the Fc region of immunoglobulins, such as FCGR1A and FCGR2A, but also CSF3R, PLAUR, the receptors for IFNG, IL2, IL6 and IL8, and integrins, such as ITGB1 and ITGB2. During the phagocytic process, mediates mobilization of secretory lysosomes, degranulation, and activation of NADPH oxidase to bring about the respiratory burst. Plays a role in the release of inflammatory molecules. Promotes reorganization of the actin cytoskeleton and actin polymerization, formation of podosomes and cell protrusions. Inhibits TP73-mediated transcription activation and TP73-mediated apoptosis. Phosphorylates CBL in response to activation of immunoglobulin gamma Fc region receptors. Phosphorylates ADAM15, BCR, ELMO1, FCGR2A, GAB1, GAB2, RAPGEF1, STAT5B, TP73, VAV1 and WAS	1.0, 5.0, 5.1, 5.2, 5.3, 5.14
HCN4	10,021	Potassium/sodium hyperpolarization-activated cyclic nucleotide-gated channel 4	Hyperpolarization-activated ion channel with very slow activation and inactivation exhibiting weak selectivity for potassium over sodium ions. Contributes to the native pacemaker currents in heart (If) that regulate the rhythm of heart beat. May contribute to the native pacemaker currents in neurons (Ih). May mediate responses to sour stimuli	3.0, 3.1, 3.6, 3.7, 3.9, 9.0
HVCN1	84,329	Voltage-gated hydrogen channel 1	Mediates the voltage-dependent proton permeability of excitable membranes. Forms a proton-selective channel through which protons may pass in accordance with their electrochemical gradient. Proton efflux, accompanied by membrane depolarization, facilitates acute production of reactive oxygen species in phagocytosis	1.0, 5.0, 5.1, 5.14
IDE	3416	Insulin-degrading enzyme	Plays a role in the cellular breakdown of insulin, APP peptides, IAPP peptides, glucagon, bradykinin, kallidin and other peptides, and thereby plays a role in intercellular peptide signaling. Substrate binding induces important conformation changes, making it possible to bind and degrade larger substrates, such as insulin. Contributes to the regulation of peptide hormone signaling cascades and regulation of blood glucose homeostasis via its role in the degradation of insulin, glucagon and IAPP (By similarity). Plays a role in the degradation and clearance of APP-derived amyloidogenic peptides that are secreted by neurons and microglia (Probable). Involved in antigen processing. Produces both the N terminus and the C terminus of MAGEA3-derived antigenic peptide (EVDPIGHLY) that is presented to cytotoxic T lymphocytes by MHC class I	1.0, 9.0
IL2RA	3559	Interleukin-2 receptor subunit alpha	Receptor for interleukin-2. The receptor is involved in the regulation of immune tolerance by controlling regulatory T cells (TREGs) activity. TREGs suppress the activation and expansion of autoreactive T-cells.", NA)	5.0, 5.1, 5.2, 5.4, 5.5, 5.8
IL5	3567	Interleukin-5	Factor that induces terminal differentiation of late-developing B-cells to immunoglobulin secreting cells	2.0, 2.9
ITGA1	3672	Integrin alpha-1	Integrin alpha-1/beta-1 is a receptor for laminin and collagen. It recognizes the proline-hydroxylated sequence G-F-P-G-E-R in collagen. Involved in anchorage-dependent, negative regulation of EGF-stimulated cell growth	1.0, 3.0, 3.1, 3.2, 3.3, 3.4, 5.0, 5.1, 5.14
KCNK10	54,207	Potassium channel subfamily K member 10	Outward rectifying potassium channel. Produces rapidly activating and non-inactivating outward rectifier K( +) currents. Activated by arachidonic acid and other naturally occurring unsaturated free fatty acids	1
KDR	3791	Vascular endothelial growth factor receptor 2	Tyrosine-protein kinase that acts as a cell-surface receptor for VEGFA, VEGFC and VEGFD. Plays an essential role in the regulation of angiogenesis, vascular development, vascular permeability, and embryonic hematopoiesis. Promotes proliferation, survival, migration and differentiation of endothelial cells. Promotes reorganization of the actin cytoskeleton. Isoforms lacking a transmembrane domain, such as isoform 2 and isoform 3, may function as decoy receptors for VEGFA, VEGFC and/or VEGFD. Isoform 2 plays an important role as negative regulator of VEGFA- and VEGFC-mediated lymphangiogenesis by limiting the amount of free VEGFA and/or VEGFC and preventing their binding to FLT4. Modulates FLT1 and FLT4 signaling by forming heterodimers. Binding of vascular growth factors to isoform 1 leads to the activation of several signaling cascades. Activation of PLCG1 leads to the production of the cellular signaling molecules diacylglycerol and inositol 1,4,5-trisphosphate and the activation of protein kinase C. Mediates activation of MAPK1/ERK2, MAPK3/ERK1 and the MAP kinase signaling pathway, as well as of the AKT1 signaling pathway. Mediates phosphorylation of PIK3R1, the regulatory subunit of phosphatidylinositol 3-kinase, reorganization of the actin cytoskeleton and activation of PTK2/FAK1. Required for VEGFA-mediated induction of NOS2 and NOS3, leading to the production of the signaling molecule nitric oxide (NO) by endothelial cells. Phosphorylates PLCG1. Promotes phosphorylation of FYN, NCK1, NOS3, PIK3R1, PTK2/FAK1 and SRC	1
KRAS	3845	V-Ki-ras2 Kirsten rat sarcoma viral oncogene homolog, isoform CRA_b	Ras proteins bind GDP/GTP and possess intrinsic GTPase activity. Plays an important role in the regulation of cell proliferation. Plays a role in promoting oncogenic events by inducing transcriptional silencing of tumor suppressor genes (TSGs) in colorectal cancer (CRC) cells in a ZNF304-dependent manner	1.0, 2.0
KRT10	3858	Keratin, type I cytoskeletal 10	Plays a role in the establishment of the epidermal barrier on plantar skin	1.0, 2.0
LEP	3952	Leptin	Key player in the regulation of energy balance and body weight control. Once released into the circulation, has central and peripheral effects by binding LEPR, found in many tissues, which results in the activation of several major signaling pathways. In the hypothalamus, acts as an appetite-regulating factor that induces a decrease in food intake and an increase in energy consumption by inducing anorexinogenic factors and suppressing orexigenic neuropeptides, also regulates bone mass and secretion of hypothalamo-pituitary-adrenal hormones. In the periphery, increases basal metabolism, influences reproductive function, regulates pancreatic beta-cell function and insulin secretion, is pro-angiogenic for endothelial cell and affects innate and adaptive immunity. In the arcuate nucleus of the hypothalamus, activates by depolarization POMC neurons inducing FOS and SOCS3 expression to release anorexigenic peptides and inhibits by hyperpolarization NPY neurons inducing SOCS3 with a consequent reduction on release of orexigenic peptides. In addition to its known satiety inducing effect, has a modulatory role in nutrient absorption. In the intestine, reduces glucose absorption by enterocytes by activating PKC and leading to a sequential activation of p38, PI3K and ERK signaling pathways which exerts an inhibitory effect on glucose absorption. Acts as a growth factor on certain tissues, through the activation of different signaling pathways increases expression of genes involved in cell cycle regulation such as CCND1, via JAK2-STAT3 pathway, or VEGFA, via MAPK1/3 and PI3K-AKT1 pathways. May also play an apoptotic role via JAK2-STAT3 pathway and up-regulation of BIRC5 expression. Pro-angiogenic, has mitogenic activity on vascular endothelial cells and plays a role in matrix remodeling by regulating the expression of matrix metalloproteinases (MMPs) and tissue inhibitors of metalloproteinases (TIMPs). In innate immunity, modulates the activity and function of neutrophils by increasing chemotaxis and the secretion of oxygen radicals. Increases phagocytosis by macrophages and enhances secretion of pro-inflammatory mediators. Increases cytotoxic ability of NK cells. Plays a pro-inflammatory role, in synergy with IL1B, by inducing NOS2 wich promotes the production of IL6, IL8 and Prostaglandin E2, through a signaling pathway that involves JAK2, PI3K, MAP2K1/MEK1 and MAPK14/p38. In adaptive immunity, promotes the switch of memory T-cells towards T helper-1 cell immune responses. Increases CD4( +)CD25(-) T-cell proliferation and reduces autophagy during TCR (T-cell receptor) stimulation, through MTOR signaling pathway activation and BCL2 up-regulation	5
LGALS2	3957	Galectin-2	This protein binds beta-galactoside. Its physiological function is not yet known	3
LIG4	3981	DNA ligase	Efficiently joins single-strand breaks in a double-stranded polydeoxynucleotide in an ATP-dependent reaction. Involved in DNA non-homologous end joining (NHEJ) required for double-strand break repair and V(D)J recombination. The LIG4-XRCC4 complex is responsible for the NHEJ ligation step, and XRCC4 enhances the joining activity of LIG4. Binding of the LIG4-XRCC4 complex to DNA ends is dependent on the assembly of the DNA-dependent protein kinase complex DNA-PK to these DNA ends	5
LRRC8A	56,262	Leucine rich repeat containing 8 family, member A, isoform CRA_a	Essential component of the volume-regulated anion channel (VRAC, also named VSOAC channel), an anion channel required to maintain a constant cell volume in response to extracellular or intracellular osmotic changes. The VRAC channel conducts iodide better than chloride and can also conduct organic osmolytes like taurine. Mediates efflux of amino acids, such as aspartate and glutamate, in response to osmotic stress. LRRC8A and LRRC8D are required for the uptake of the drug cisplatin. Required for in vivo channel activity, together with at least one other family member (LRRC8B, LRRC8C, LRRC8D or LRRC8E); channel characteristics depend on the precise subunit composition. Can form functional channels by itself (in vitro). Involved in B-cell development: required for the pro-B cell to pre-B cell transition. Also required for T-cell development (By similarity). May play a role in lysosome homeostasis	5
LTA	4049	Lymphotoxin-alpha	Cytokine that in its homotrimeric form binds to TNFRSF1A/TNFR1, TNFRSF1B/TNFBR and TNFRSF14/HVEM. In its heterotrimeric form with LTB binds to TNFRSF3/LTBR. Lymphotoxin is produced by lymphocytes and is cytotoxic for a wide range of tumor cells in vitro and in vivo.", NA)	3.0, 5.0
LTF	4057	Lactotransferrin	[Isoform DeltaLf]: transcription factor with antiproliferative properties and ability to induce cell cycle arrest. Binds to the DeltaLf response element found in the SKP1, BAX, DCPS, and SELENOH promoters	5
MAPK8IP3	23,162	C-Jun-amino-terminal kinase-interacting protein 3	The JNK-interacting protein (JIP) group of scaffold proteins selectively mediates JNK signaling by aggregating specific components of the MAPK cascade to form a functional JNK signaling module. May function as a regulator of vesicle transport, through interactions with the JNK-signaling components and motor proteins (By similarity). Promotes neuronal axon elongation in a kinesin- and JNK-dependent manner. Activates cofilin at axon tips via local activation of JNK, thereby regulating filopodial dynamics and enhancing axon elongation. Its binding to kinesin heavy chains (KHC), promotes kinesin-1 motility along microtubules and is essential for axon elongation and regeneration. Regulates cortical neuronal migration by mediating NTRK2/TRKB anterograde axonal transport during brain development (By similarity). Acts as an adapter that bridges the interaction between NTRK2/TRKB and KLC1 and drives NTRK2/TRKB axonal but not dendritic anterograde transport, which is essential for subsequent BDNF-triggered signaling and filopodia formation	5
MELK	9833	Non-specific serine/threonine protein kinase	Serine/threonine-protein kinase involved in various processes such as cell cycle regulation, self-renewal of stem cells, apoptosis and splicing regulation. Has a broad substrate specificity; phosphorylates BCL2L14, CDC25B, MAP3K5/ASK1 and ZNF622. Acts as an activator of apoptosis by phosphorylating and activating MAP3K5/ASK1. Acts as a regulator of cell cycle, notably by mediating phosphorylation of CDC25B, promoting localization of CDC25B to the centrosome and the spindle poles during mitosis. Plays a key role in cell proliferation and carcinogenesis. Required for proliferation of embryonic and postnatal multipotent neural progenitors. Phosphorylates and inhibits BCL2L14, possibly leading to affect mammary carcinogenesis by mediating inhibition of the pro-apoptotic function of BCL2L14. Also involved in the inhibition of spliceosome assembly during mitosis by phosphorylating ZNF622, thereby contributing to its redirection to the nucleus. May also play a role in primitive hematopoiesis	general; no clear pathology
MFN2	9927	Mitofusin-2	Mitochondrial outer membrane GTPase that mediates mitochondrial clustering and fusion. Mitochondria are highly dynamic organelles, and their morphology is determined by the equilibrium between mitochondrial fusion and fission events. Overexpression induces the formation of mitochondrial networks. Membrane clustering requires GTPase activity and may involve a major rearrangement of the coiled coil domains (Probable). Plays a central role in mitochondrial metabolism and may be associated with obesity and/or apoptosis processes (By similarity). Plays an important role in the regulation of vascular smooth muscle cell proliferation (By similarity). Involved in the clearance of damaged mitochondria via selective autophagy (mitophagy). Is required for PRKN recruitment to dysfunctional mitochondria. Involved in the control of unfolded protein response (UPR) upon ER stress including activation of apoptosis and autophagy during ER stress (By similarity). Acts as an upstream regulator of EIF2AK3 and suppresses EIF2AK3 activation under basal conditions (By similarity)	9
MMP1	4312	cDNA FLJ55228, highly similar to Interstitial collagenase	Cleaves collagens of types I, II, and III at one site in the helical domain. Also cleaves collagens of types VII and X. In case of HIV infection, interacts and cleaves the secreted viral Tat protein, leading to a decrease in neuronal Tat's mediated neurotoxicity	2
MSN	4478	Moesin	Ezrin-radixin-moesin (ERM) family protein that connects the actin cytoskeleton to the plasma membrane and thereby regulates the structure and function of specific domains of the cell cortex. Tethers actin filaments by oscillating between a resting and an activated state providing transient interactions between moesin and the actin cytoskeleton. Once phosphorylated on its C-terminal threonine, moesin is activated leading to interaction with F-actin and cytoskeletal rearrangement. These rearrangements regulate many cellular processes, including cell shape determination, membrane transport, and signal transduction. The role of moesin is particularly important in immunity acting on both T and B-cells homeostasis and self-tolerance, regulating lymphocyte egress from lymphoid organs. Modulates phagolysosomal biogenesis in macrophages (By similarity). Participates also in immunologic synapse formation.", NA)	5
MTHFR	4524	Methylenetetrahydrofolate reductase	Catalyzes the conversion of 5,10-methylenetetrahydrofolate to 5-methyltetrahydrofolate, a co-substrate for homocysteine remethylation to methionine.", NA)	4.0, 4.4, 4.5, 4.8, 4.12
MYDGF	56,005	Myeloid-derived growth factor	Bone marrow-derived monocyte and paracrine-acting protein that promotes cardiac myocyte survival and adaptive angiogenesis for cardiac protection and/or repair after myocardial infarction (MI). Stimulates endothelial cell proliferation through a MAPK1/3-, STAT3- and CCND1-mediated signaling pathway. Inhibits cardiac myocyte apoptosis in a PI3K/AKT-dependent signaling pathway (By similarity). Involved in endothelial cell proliferation and angiogenesis	3
NCKIPSD	51,517	NCK-interacting protein with SH3 domain	Has an important role in stress fiber formation induced by active diaphanous protein homolog 1 (DRF1). Induces microspike formation, in vivo (By similarity). In vitro, stimulates N-WASP-induced ARP2/3 complex activation in the absence of CDC42 (By similarity). May play an important role in the maintenance of sarcomeres and/or in the assembly of myofibrils into sarcomeres. Implicated in regulation of actin polymerization and cell adhesion. Plays a role in angiogenesis	9
NPC1	4864	NPC intracellular cholesterol transporter 1	Intracellular cholesterol transporter which acts in concert with NPC2 and plays an important role in the egress of cholesterol from the endosomal/lysosomal compartment. Unesterified cholesterol that has been released from LDLs in the lumen of the late endosomes/lysosomes is transferred by NPC2 to the cholesterol-binding pocket in the N-terminal domain of NPC1. Cholesterol binds to NPC1 with the hydroxyl group buried in the binding pocket. Binds oxysterol with higher affinity than cholesterol. May play a role in vesicular trafficking in glia, a process that may be crucial for maintaining the structural and functional integrity of nerve terminals (Probable)	9.0, 9.17
NTRK1	4914	High affinity nerve growth factor receptor	Receptor tyrosine kinase involved in the development and the maturation of the central and peripheral nervous systems through regulation of proliferation, differentiation and survival of sympathetic and nervous neurons. High affinity receptor for NGF which is its primary ligand. Can also bind and be activated by NTF3/neurotrophin-3. However, NTF3 only supports axonal extension through NTRK1 but has no effect on neuron survival (By similarity). Upon dimeric NGF ligand-binding, undergoes homodimerization, autophosphorylation and activation. Recruits, phosphorylates and/or activates several downstream effectors including SHC1, FRS2, SH2B1, SH2B2 and PLCG1 that regulate distinct overlapping signaling cascades driving cell survival and differentiation. Through SHC1 and FRS2 activates a GRB2-Ras-MAPK cascade that regulates cell differentiation and survival. Through PLCG1 controls NF-Kappa-B activation and the transcription of genes involved in cell survival. Through SHC1 and SH2B1 controls a Ras-PI3 kinase-AKT1 signaling cascade that is also regulating survival. In absence of ligand and activation, may promote cell death, making the survival of neurons dependent on trophic factors	9
OCLN	100,506,658	Occludin	May play a role in the formation and regulation of the tight junction (TJ) paracellular permeability barrier	1.0, 1.6, 2.0, 8.0, 9.0
PAH	5053	Phe-4-monooxygenase	Catalyzes the hydroxylation of L-phenylalanine to L-tyrosine	9
PDCD1	5133	Programmed cell death 1 protein	Inhibitory receptor on antigen activated T-cells that plays a critical role in induction and maintenance of immune tolerance to self. Delivers inhibitory signals upon binding to ligands CD274/PDCD1L1 and CD273/PDCD1LG2. Following T-cell receptor (TCR) engagement, PDCD1 associates with CD3-TCR in the immunological synapse and directly inhibits T-cell activation (By similarity). Suppresses T-cell activation through the recruitment of PTPN11/SHP-2: following ligand-binding, PDCD1 is phosphorylated within the ITSM motif, leading to the recruitment of the protein tyrosine phosphatase PTPN11/SHP-2 that mediates dephosphorylation of key TCR proximal signaling molecules, such as ZAP70, PRKCQ/PKCtheta and CD247/CD3zeta (By similarity)	3.0, 5.0
PDPK1	5170	3-phosphoinositide-dependent protein kinase 1	Serine/threonine kinase which acts as a master kinase, phosphorylating and activating a subgroup of the AGC family of protein kinases. Its targets include: protein kinase B (PKB/AKT1, PKB/AKT2, PKB/AKT3), p70 ribosomal protein S6 kinase (RPS6KB1), p90 ribosomal protein S6 kinase (RPS6KA1, RPS6KA2 and RPS6KA3), cyclic AMP-dependent protein kinase (PRKACA), protein kinase C (PRKCD and PRKCZ), serum and glucocorticoid-inducible kinase (SGK1, SGK2 and SGK3), p21-activated kinase-1 (PAK1), protein kinase PKN (PKN1 and PKN2). Plays a central role in the transduction of signals from insulin by providing the activating phosphorylation to PKB/AKT1, thus propagating the signal to downstream targets controlling cell proliferation and survival, as well as glucose and amino acid uptake and storage. Negatively regulates the TGF-beta-induced signaling by: modulating the association of SMAD3 and SMAD7 with TGF-beta receptor, phosphorylating SMAD2, SMAD3, SMAD4 and SMAD7, preventing the nuclear translocation of SMAD3 and SMAD4 and the translocation of SMAD7 from the nucleus to the cytoplasm in response to TGF-beta. Activates PPARG transcriptional activity and promotes adipocyte differentiation. Activates the NF-kappa-B pathway via phosphorylation of IKKB. The tyrosine phosphorylated form is crucial for the regulation of focal adhesions by angiotensin II. Controls proliferation, survival, and growth of developing pancreatic cells. Participates in the regulation of Ca(2 +) entry and Ca(2 +)-activated K( +) channels of mast cells. Essential for the motility of vascular endothelial cells (ECs) and is involved in the regulation of their chemotaxis. Plays a critical role in cardiac homeostasis by serving as a dual effector for cell survival and beta-adrenergic response. Plays an important role during thymocyte development by regulating the expression of key nutrient receptors on the surface of pre-T cells and mediating Notch-induced cell growth and proliferative responses. Provides negative feedback inhibition to toll-like receptor-mediated NF-kappa-B activation in macrophages. Isoform 3 is catalytically inactive	3.0, 5.0
PIK3CG	5294	Phosphatidylinositol-4,5-bisphosphate 3-kinase	Phosphoinositide-3-kinase (PI3K) that phosphorylates PtdIns(4,5)P2 (Phosphatidylinositol 4,5-bisphosphate) to generate phosphatidylinositol 3,4,5-trisphosphate (PIP3). PIP3 plays a key role by recruiting PH domain-containing proteins to the membrane, including AKT1 and PDPK1, activating signaling cascades involved in cell growth, survival, proliferation, motility and morphology. Links G-protein coupled receptor activation to PIP3 production. Involved in immune, inflammatory and allergic responses. Modulates leukocyte chemotaxis to inflammatory sites and in response to chemoattractant agents. May control leukocyte polarization and migration by regulating the spatial accumulation of PIP3 and by regulating the organization of F-actin formation and integrin-based adhesion at the leading edge. Controls motility of dendritic cells. Together with PIK3CD is involved in natural killer (NK) cell development and migration towards the sites of inflammation. Participates in T-lymphocyte migration. Regulates T-lymphocyte proliferation and cytokine production. Together with PIK3CD participates in T-lymphocyte development. Required for B-lymphocyte development and signaling. Together with PIK3CD participates in neutrophil respiratory burst. Together with PIK3CD is involved in neutrophil chemotaxis and extravasation. Together with PIK3CB promotes platelet aggregation and thrombosis. Regulates alpha-IIb/beta-3 integrins (ITGA2B/ ITGB3) adhesive function in platelets downstream of P2Y12 through a lipid kinase activity-independent mechanism. May have also a lipid kinase activity-dependent function in platelet aggregation. Involved in endothelial progenitor cell migration. Negative regulator of cardiac contractility. Modulates cardiac contractility by anchoring protein kinase A (PKA) and PDE3B activation, reducing cAMP levels. Regulates cardiac contractility also by promoting beta-adrenergic receptor internalization by binding to GRK2 and by non-muscle tropomyosin phosphorylation. Also has serine/threonine protein kinase activity: both lipid and protein kinase activities are required for beta-adrenergic receptor endocytosis. May also have a scaffolding role in modulating cardiac contractility. Contributes to cardiac hypertrophy under pathological stress. Through simultaneous binding of PDE3B to RAPGEF3 and PIK3R6 is assembled in a signaling complex in which the PI3K gamma complex is activated by RAPGEF3 and which is involved in angiogenesis	3.0, 4.0, 5.0
PRKG1	5592	cGMP-dependent protein kinase 1	Serine/threonine protein kinase that acts as key mediator of the nitric oxide (NO)/cGMP signaling pathway. GMP binding activates PRKG1, which phosphorylates serines and threonines on many cellular proteins. Numerous protein targets for PRKG1 phosphorylation are implicated in modulating cellular calcium, but the contribution of each of these targets may vary substantially among cell types. Proteins that are phosphorylated by PRKG1 regulate platelet activation and adhesion, smooth muscle contraction, cardiac function, gene expression, feedback of the NO-signaling pathway, and other processes involved in several aspects of the CNS like axon guidance, hippocampal and cerebellar learning, circadian rhythm and nociception. Smooth muscle relaxation is mediated through lowering of intracellular free calcium, by desensitization of contractile proteins to calcium, and by decrease in the contractile state of smooth muscle or in platelet activation. Regulates intracellular calcium levels via several pathways: phosphorylates IRAG1 and inhibits IP3-induced Ca(2 +) release from intracellular stores, phosphorylation of KCNMA1 (BKCa) channels decreases intracellular Ca(2 +) levels, which leads to increased opening of this channel. PRKG1 phosphorylates the canonical transient receptor potential channel (TRPC) family which inactivates the associated inward calcium current. Another mode of action of NO/cGMP/PKGI signaling involves PKGI-mediated inactivation of the Ras homolog gene family member A (RhoA). Phosphorylation of RHOA by PRKG1 blocks the action of this protein in myriad processes: regulation of RHOA translocation; decreasing contraction; controlling vesicle trafficking, reduction of myosin light chain phosphorylation resulting in vasorelaxation. Activation of PRKG1 by NO signaling alters also gene expression in a number of tissues. In smooth muscle cells, increased cGMP and PRKG1 activity influence expression of smooth muscle-specific contractile proteins, levels of proteins in the NO/cGMP signaling pathway, down-regulation of the matrix proteins osteopontin and thrombospondin-1 to limit smooth muscle cell migration and phenotype. Regulates vasodilator-stimulated phosphoprotein (VASP) functions in platelets and smooth muscle	9.5; 9.9; 9.10; 4.8; 4.5; 4.4; 3.12; 3.13; 3.7; 3.6
PTPN23	25,930	cDNA FLJ58078, highly similar to Tyrosine-protein phosphatase non-receptortype 23	Plays a role in sorting of endocytic ubiquitinated cargos into multivesicular bodies (MVBs) via its interaction with the ESCRT-I complex (endosomal sorting complex required for transport I), and possibly also other ESCRT complexes. May act as a negative regulator of Ras-mediated mitogenic activity. Plays a role in ciliogenesis	8.0, 8.1, 9.0, 9.12, 9.13, 9.16,
RASGRP1	10,125	cDNA, FLJ94771, highly similar to Homo sapiens RAS guanyl releasing protein 1 , mRNA	Functions as a calcium- and diacylglycerol (DAG)-regulated nucleotide exchange factor specifically activating Ras through the exchange of bound GDP for GTP. Activates the Erk/MAP kinase cascade. Regulates T-cell/B-cell development, homeostasis and differentiation by coupling T-lymphocyte/B-lymphocyte antigen receptors to Ras. Regulates NK cell cytotoxicity and ITAM-dependent cytokine production by activation of Ras-mediated ERK and JNK pathways. Functions in mast cell degranulation and cytokine secretion, regulating FcERI-evoked allergic responses. May also function in differentiation of other cell types	5.0, 5.1, 5.7, 5.8, 5.14
ROCK1	6093	Rho-associated protein kinase 1	Protein kinase which is a key regulator of actin cytoskeleton and cell polarity. Involved in regulation of smooth muscle contraction, actin cytoskeleton organization, stress fiber and focal adhesion formation, neurite retraction, cell adhesion and motility via phosphorylation of DAPK3, GFAP, LIMK1, LIMK2, MYL9/MLC2, TPPP, PFN1 and PPP1R12A. Phosphorylates FHOD1 and acts synergistically with it to promote SRC-dependent non-apoptotic plasma membrane blebbing. Phosphorylates JIP3 and regulates the recruitment of JNK to JIP3 upon UVB-induced stress. Acts as a suppressor of inflammatory cell migration by regulating PTEN phosphorylation and stability (By similarity). Acts as a negative regulator of VEGF-induced angiogenic endothelial cell activation. Required for centrosome positioning and centrosome-dependent exit from mitosis (By similarity). Plays a role in terminal erythroid differentiation. May regulate closure of the eyelids and ventral body wall by inducing the assembly of actomyosin bundles (By similarity). Promotes keratinocyte terminal differentiation. Involved in osteoblast compaction through the fibronectin fibrillogenesis cell-mediated matrix assembly process, essential for osteoblast mineralization (By similarity)	1.0, 1.1, 2.0, 2.8, 3.0, 3.6, 3.7, 3.12, 4.0, 4.5, 9.0, 9.10, 9.17
SH3GL2	6456	Uncharacterized protein DKFZp686B23205	Implicated in synaptic vesicle endocytosis. May recruit other proteins to membranes with high curvature. Required for BDNF-dependent dendrite outgrowth. Cooperates with SH3GL2 to mediate BDNF-NTRK2 early endocytic trafficking and signaling from early endosomes	9.0, 9.5, 9.16
SLK	9748	STE20-like serine/threonine-protein kinase	Mediates apoptosis and actin stress fiber dissolution	4.0, 4.2, 7.0, 7.1, 7.2, 5.14
SNAP23	8773	Synaptosomal-associated protein, 23 kDa, isoform CRA_b	Essential component of the high affinity receptor for the general membrane fusion machinery and an important regulator of transport vesicle docking and fusion	4.0, 4.2, 4.3, 4.5, 8.1, 8.2
SNX33	257,364	Sorting nexin-33	Plays a role in the reorganization of the cytoskeleton, endocytosis and cellular vesicle trafficking via its interactions with membranes, WASL, DNM1 and DNM2. Acts both during interphase and at the end of mitotic cell divisions. Required for efficient progress through mitosis and cytokinesis. Required for normal formation of the cleavage furrow at the end of mitosis. Modulates endocytosis of cell-surface proteins, such as APP and PRNP; this then modulates the secretion of APP and PRNP peptides. Promotes membrane tubulation (in vitro). May promote the formation of macropinosomes	9.0, 9.5, 9.16
SPHK1	8877	Sphingosine kinase 1	Catalyzes the phosphorylation of sphingosine to form sphingosine 1-phosphate (SPP), a lipid mediator with both intra- and extracellular functions. Also acts on D-erythro-sphingosine and to a lesser extent sphinganine, but not other lipids, such as D,L-threo-dihydrosphingosine, N,N-dimethylsphingosine, diacylglycerol, ceramide, or phosphatidylinositol. In contrast to proapoptotic SPHK2, has a negative effect on intracellular ceramide levels, enhances cell growth and inhibits apoptosis. Involved in the regulation of inflammatory response and neuroinflammation. Via the product sphingosine 1-phosphate, stimulates TRAF2 E3 ubiquitin ligase activity, and promotes activation of NF-kappa-B in response to TNF signaling leading to IL17 secretion. In response to TNF and in parallel to NF-kappa-B activation, negatively regulates RANTES induction through p38 MAPK signaling pathway. Involved in endocytic membrane trafficking induced by sphingosine, recruited to dilate endosomes, also plays a role on later stages of endosomal maturation and membrane fusion independently of its kinase activity. In Purkinje cells, seems to be also involved in the regulation of autophagosome-lysosome fusion upon VEGFA	6.0, 6.2, 6.3, 8.0, 8.1, 8.2, 5.0, 5.2, 9.0, 9.1, 9.4
SPR	6697	Sepiapterin reductase	Catalyzes the final one or two reductions in tetra-hydrobiopterin biosynthesis to form 5,6,7,8-tetrahydrobiopterin	9.0, 9.1, 9.5, 9.9, 9.17
STIM1	6786	STIM1L	Plays a role in mediating store-operated Ca(2 +) entry (SOCE), a Ca(2 +) influx following depletion of intracellular Ca(2 +) stores. Acts as Ca(2 +) sensor in the endoplasmic reticulum via its EF-hand domain. Upon Ca(2 +) depletion, translocates from the endoplasmic reticulum to the plasma membrane where it activates the Ca(2 +) release-activated Ca(2 +) (CRAC) channel subunit ORAI1. Involved in enamel formation. Activated following interaction with STIMATE, leading to promote STIM1 conformational switch	3.0, 3.11, 4.0, 4,1, 4.5, 5.0, 5.13, 5.14, 8.0, 8.1, 8.2
STK17B	9262	Serine/threonine-protein kinase 17B	Phosphorylates myosin light chains (By similarity). Acts as a positive regulator of apoptosis.", NA)	1.0, 1.8, 1.9, 2.0, 2.8, 2.10, 2.11
THOP1	7064	Thimet oligopeptidase	Involved in the metabolism of neuropeptides under 20 amino acid residues long. Involved in cytoplasmic peptide degradation. Able to degrade the amyloid-beta precursor protein and generate amyloidogenic fragments	9.0, 9.12, 9.16, 5.0, 5.1, 5.2, 5.14
TNK2	10,188	Non-specific protein-tyrosine kinase	Non-receptor tyrosine-protein and serine/threonine-protein kinase that is implicated in cell spreading and migration, cell survival, cell growth and proliferation. Transduces extracellular signals to cytosolic and nuclear effectors. Phosphorylates AKT1, AR, MCF2, WASL and WWOX. Implicated in trafficking and clathrin-mediated endocytosis through binding to epidermal growth factor receptor (EGFR) and clathrin. Binds to both poly- and mono-ubiquitin and regulates ligand-induced degradation of EGFR, thereby contributing to the accumulation of EGFR at the limiting membrane of early endosomes. Downstream effector of CDC42 which mediates CDC42-dependent cell migration via phosphorylation of BCAR1. May be involved both in adult synaptic function and plasticity and in brain development. Activates AKT1 by phosphorylating it on 'Tyr-176'. Phosphorylates AR on 'Tyr-267' and 'Tyr-363' thereby promoting its recruitment to androgen-responsive enhancers (AREs). Phosphorylates WWOX on 'Tyr-287'. Phosphorylates MCF2, thereby enhancing its activity as a guanine nucleotide exchange factor (GEF) toward Rho family proteins. Contributes to the control of AXL receptor levels. Confers metastatic properties on cancer cells and promotes tumor growth by negatively regulating tumor suppressor such as WWOX and positively regulating pro-survival factors such as AKT1 and AR. Phosphorylates WASP	1.0, 1.1, 1.6, 1.8, 2.0, 2.11, 8.0, 8.2, 8.3, 8.4, 9.0, 9.13,9.17
USP14	9097	Ubiquitin carboxyl-terminal hydrolase 14	Proteasome-associated deubiquitinase which releases ubiquitin from the proteasome targeted ubiquitinated proteins. Ensures the regeneration of ubiquitin at the proteasome. Is a reversibly associated subunit of the proteasome and a large fraction of proteasome-free protein exists within the cell. Required for the degradation of the chemokine receptor CXCR4 which is critical for CXCL12-induced cell chemotaxis. Serves also as a physiological inhibitor of endoplasmic reticulum-associated degradation (ERAD) under the non-stressed condition by inhibiting the degradation of unfolded endoplasmic reticulum proteins via interaction with ERN1. Indispensable for synaptic development and function at neuromuscular junctions (NMJs). Plays a role in the innate immune defense against viruses by stabilizing the viral DNA sensor CGAS and thus inhibiting its autophagic degradation	3.0, 3.2, 3.8, 3.9, 3.11, 5.0, 5.1, 5.13, 5.14
USP8	9101	Ubiquitin specific peptidase 8, isoform CRA_a	Hydrolase that can remove conjugated ubiquitin from proteins and therefore plays an important regulatory role at the level of protein turnover by preventing degradation. Converts both 'Lys-48' an 'Lys-63'-linked ubiquitin chains. Catalytic activity is enhanced in the M phase. Involved in cell proliferation. Required to enter into S phase in response to serum stimulation. May regulate T-cell anergy mediated by RNF128 via the formation of a complex containing RNF128 and OTUB1. Probably regulates the stability of STAM2 and RASGRF1. Regulates endosomal ubiquitin dynamics, cargo sorting, membrane traffic at early endosomes, and maintenance of ESCRT-0 stability. The level of protein ubiquitination on endosomes is essential for maintaining the morphology of the organelle. Deubiquitinates EPS15 and controles tyrosine kinase stability. Removes conjugated ubiquitin from EGFR thus regulating EGFR degradation and downstream MAPK signaling. Involved in acrosome biogenesis through interaction with the spermatid ESCRT-0 complex and microtubules. Deubiquitinates BIRC6/bruce and KIF23/MKLP1. Deubiquitinates BACE1 which inhibits BACE1 lysosomal degradation and modulates BACE-mediated APP cleavage and amyloid-beta formation	5.0, 5.1, 5.2, 5.3, 7.0, 7.1, 7.2
VCL	7414	Metavinculin	Actin filament (F-actin)-binding protein involved in cell–matrix adhesion and cell–cell adhesion. Regulates cell-surface E-cadherin expression and potentiates mechanosensing by the E-cadherin complex. May also play important roles in cell morphology and locomotion	3.0, 3.1, 3.2, 4.0, 4.5, 7.0, 7.1, 7.2
VEGFA	7422	Vascular endothelial growth factor A	Growth factor active in angiogenesis, vasculogenesis and endothelial cell growth. Induces endothelial cell proliferation, promotes cell migration, inhibits apoptosis and induces permeabilization of blood vessels. Binds to the FLT1/VEGFR1 and KDR/VEGFR2 receptors, heparan sulfate and heparin. NRP1/Neuropilin-1 binds isoforms VEGF-165 and VEGF-145. Isoform VEGF165B binds to KDR but does not activate downstream signaling pathways, does not activate angiogenesis and inhibits tumor growth. Binding to NRP1 receptor initiates a signaling pathway needed for motor neuron axon guidance and cell body migration, including for the caudal migration of facial motor neurons from rhombomere 4 to rhombomere 6 during embryonic development (By similarity)	1.0, 1.1, 1.8, 1.11, 2.0, 2.10, 2.11, 3.0, 3.9, 4.0, 4.2, 4.5, 5.0, 5.14, 6.0, 6.3, 7.0, 7.1, 7.2, 8.0, 8.1, 8.2, 8.3, 8.4, 9.0, 9.7, 9.10, 9.17
XDH	7498	Xanthine dehydrogenase/oxidase	Key enzyme in purine degradation. Catalyzes the oxidation of hypoxanthine to xanthine. Catalyzes the oxidation of xanthine to uric acid. Contributes to the generation of reactive oxygen species. Has also low oxidase activity towards aldehydes (in vitro)	1.0, 1.1, 1.4, 1.7, 1.8, 1.10, 1.11, 2.0, 2.11, 2.17, 3.0, 3.9, 3.11, 3.13, 4.0, 4.5, 5.0, 5.14, 6.0, 6.2, 6.3, 7.0, 7.1, 7.2, 8.0, 8.1, 8.2, 8.3, 8.4, 9.0, 9.4, 9.13, 9.10, 9.17

### The biology of human structural homologues of SARS-CoV-2

**Figure 3:**
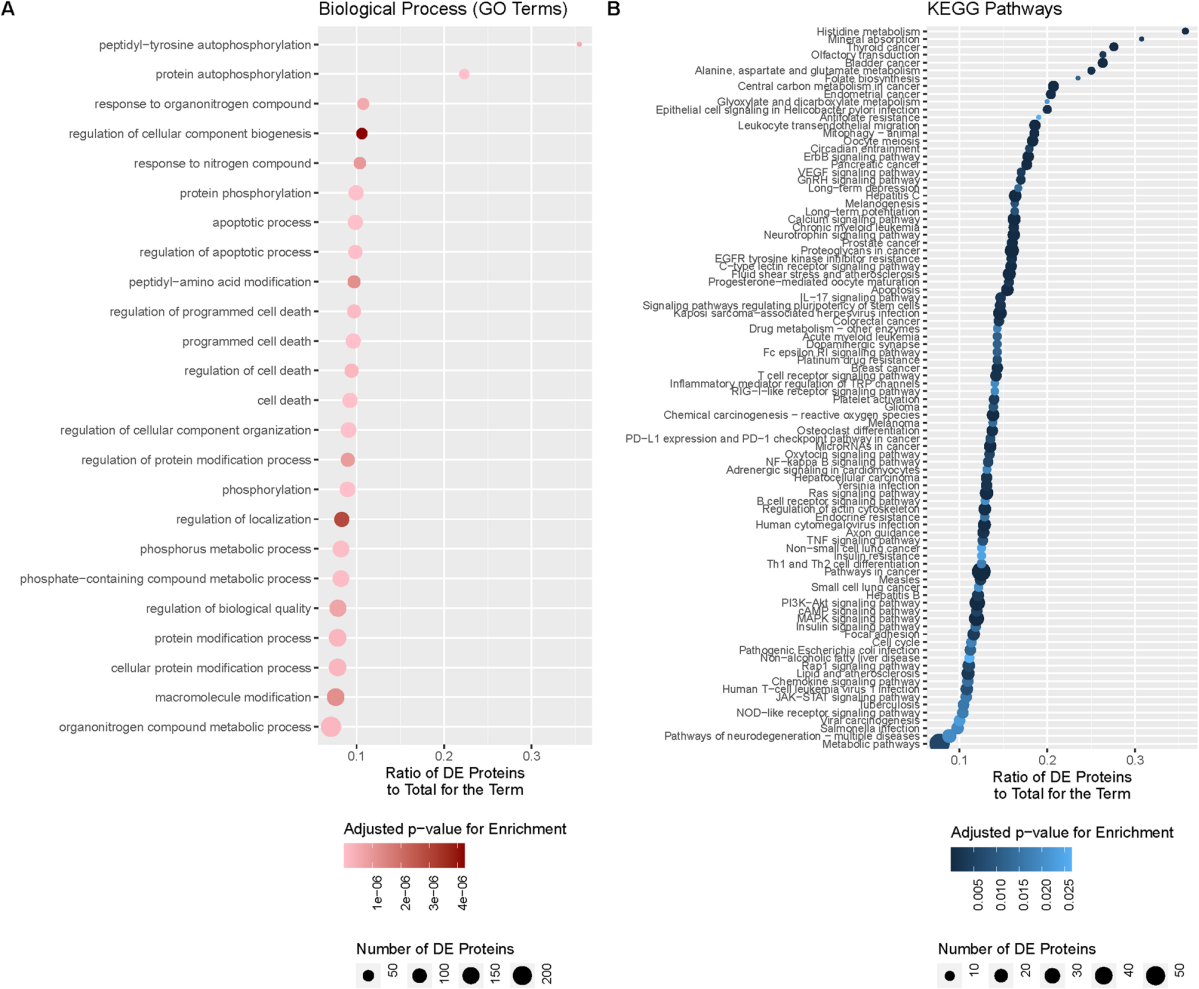
Biological processes related to predicted homologous proteins. Over enriched GO terms **(A)** and over enriched KEGG Pathways **(B)** are shown here. These show which terms and pathways are overrepresented in our set of 346 proteins which have significant structural homology to a SARS-CoV-2 protein. Terms with adjusted p-values less than 0.05 are shown here. The size of each dot shows the number of proteins represented in our data set which fall in that category. The color of the dot shows the adjusted p-value of the enrichment.

**Figure 4:**
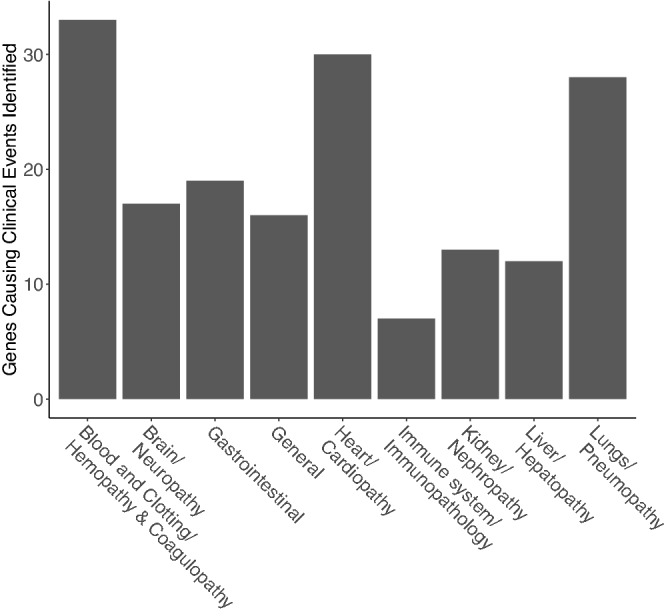
An overview of clinical indications. The distribution of top-level Clinical Indication shows that proteins with structural homologies span a wide range of clinical indication.

We have identified the Gene Ontology (GO) Terms and Kyoto Encyclopedia of Genes and Genomes (KEGG) Pathways enriched by the list of 346 structurally homologous proteins (Fig. 3)^27,32,33^. Based on literature searches we have listed the biological functions of the 102 human proteins which show structural homology with SARS-CoV-2 proteins. These proteins exhibit diverse biological functions and could thus potentially affect multiple biochemical pathways (Table 2, Fig. 4).

## Discussion

Immunodominant antigens introduced by pathogens have the potential to trigger cross-reactive immune responses that may impact the function of bystander endogenous host proteins with shared structural epitopes and result in an autoimmune disease. Some examples of cross-reactive immune responses following viral infection include type 1 diabetes mellitus [coxsackievirus; cytomegalovirus; enterovirus], multiple sclerosis [cytomegalovirus; Epstein Barr virus; measles virus; Theiler’s virus; Varicella-Zoster virus; West Nile virus], immune thrombocytopenia [Hepatitis C virus], myasthenia gravis [West Nile virus], Guillain-Barré syndrome [Zika virus], and rheumatoid arthritis [cytomegalovirus; Epstein-Barr virus]^34–36^.

Since the first COVID-19 cases were identified, a diverse array of clinical indications have been reported to be associated with SARS-CoV-2 infections (Table 1). Although SARS-CoV-2 is primarily a respiratory pathogen, there can be multiple organ systems involvement. Thus, hematological, cardiovascular, neurological and gastrointestinal complications have been reported in COVID-19 patients^37–44^. Many of these pathologies are difficult to explain on the basis of the route of entry and/or sites of SARS-CoV-2 infection. In this theoretical study we have explored the hypothesis that cross-reactivity of antibodies that target the SARS-CoV-2 proteins to endogenous human proteins may play a role in at least some of the dizzying array of clinical presentations of COVID-19 patients. Clinical data suggest that this is a plausible hypothesis to investigate. A prospective study involving 22 German patients suggests that SARS-CoV-2 infection could elicit organ specific autoimmunity in susceptible patients and lead to respiratory failure^45^. Similarly, a retrospective study of 21 patients with critical SARS-CoV-2 pneumonia, detected autoantibodies related to autoimmune disease^17^. In one study, a high-throughput auto-antibody detection technique was applied on 194 SARS-CoV-2-positive subjects^20^. These subjects showed higher levels of autoantibodies to diverse human antigens compared to controls who were SARS-CoV-2 negative.

Several in silico approaches have been used to identify and study potential cross-reactivity between pathogen-derived and human proteins^12,13^. These studies primarily rely on identifying amino acid sequence homologies between proteins from the pathogen and endogenous human proteins. In subsequent analysis some of the studies have endeavored to then determine which (if any) of these homologous regions are potential T cell epitopes based on their affinities for HLA class I and class II alleles^30,31^. We on the other hand, rationalized that anti-SARS-CoV-2 antibodies that cross-react with endogenous human proteins and elicit auto-immune pathologies are more likely to interact with conformational domains. However, using computational tools to compare conformational homologies between host and pathogen proteins is far more challenging than carrying out a sequence homology. We have limited our search to proteins which have a known structure and PDB file and used the ProBiS algorithm^22,23^ to match surface patches on chains of human proteins to surface patches on SARS-CoV-2 proteins [See “Materials and methods”]. Using this method, we have identified 346 human proteins that show structural homology to SARS-CoV-2 viral antigens (Supplemental Table 4).

We concomitantly identified proteins that are linked to clinical conditions or symptoms reported for COVID-19. We carried out an in-depth literature survey to record and classify many clinical manifestations reported in patients diagnosed with COVID-19 (Table 1). We also used a numeric notation for each clinical condition to allow cross-referencing. Our analysis shows that of the 346 human proteins that showed structural homology to SARS-CoV-2, 102 proteins have biological functions which, if disrupted, could result in pathologies associated with COVID-19 (the pathologies and proteins are depicted in Tables 1 and 2). We again emphasize that these 102 human genes have not been experimentally verified but provide a data set which could provide a useful resource. This list could be a starting point for carrying out in vitro studies to elucidate the mechanistic basis of clinical observations.

We have identified human proteins with structural homology to SARS CoV-2 proteins that, if functionally inhibited (e.g., by cross-reactive anti-SARS CoV-2 antibodies), may be mechanistically implicated in the development of severe COVID-19 clinical manifestations. An exhaustive discussion of each candidate human protein and its possible implication to COVID-19 clinical presentation is not possible. However, we discuss several examples of identified candidate proteins and their possible connection to severe COVID-19 illness to illustrate the potential, practical utility of our theoretical study and resultant data sets. Some genes that may be related to severe COVID-19 pathophysiology and are good candidates for experimental investigation include PRKG1, ACE, CFB, CRP, CTNNB1, EGFR, and VEGFA.

The PRKG1 gene product, protein kinase G1 (PKG-1), is serine/threonine-specific protein kinase that is activated by Cyclic guanosine monophosphate (cGMP) 2. PKG1-1 regulates vascular smooth muscle relaxation and modulates the contractility, growth, and apoptosis of cardiomyocytes^46–49^. Involvement of the cGMP-PKG signaling pathway in the cardiac contractility makes the PRKG1 gene a possible candidate for heart failure in COVID-19 patients.

The renin-angiotensin system (RAS) is known for its effects on the cardiovascular system and fluid hemostasis^50^. Increased activity of the vasoconstrictive and proliferative axis such as angiotensin II/ Angiotensin-converting enzyme (ACE)/ AT1 has been reported to be associated with a higher risk of acute thrombosis through the destabilizing of atherosclerotic plaque and enhancing the platelet activity and coagulation^51^. ACE2 shares 40% identity and 61% similarity with ACE^52^. SARS-Cov-2 infection mediated by ACE2 and TMPSRSS2 proteins is well established^53^. ACE2 is expressed in cells from multiple tissues, including airways, cornea, esophagus, ileum, colon, liver, gallbladder, heart, kidney and testis^54^. Similarly, to SARS-CoV^55^ infection with SARS-CoV-2 may downregulate cell surface expression of ACE2 and may result in reduced activity of ACE2 in infected organs. Moreover, binding of ACE2 to SARS-CoV, and most likely with SARS-CoV-2, increases the activity of disintegrin and metalloproteinase domain-containing protein (ADAMTS17)^57^ which can induce the shedding of ectodomain form of ACE2 and detectable the soluble ACE2^58^. The shedding of myocardial ACE2 into the circulation and its association with heart disease in preclinical models suggest that the loss of tissue ACE2 plays a pathogenic role in heart disease^59,60^. Varying ACE2 expression might affect disease susceptibility and progression. Generally, ACE2 expression is highest in children, young people, and women, decreases with age and is lowest in people with underlying conditions such as diabetes and hypertension. Therefore, lower levels of expression of the viral receptor ACE2 are found in those at the highest risk for progression of COVID-19 to a severe disease phenotype^61,61^.

The liver is the major site of complement synthesis. Complement factor B is a protein encoded by the CFB gene. Complement factor B generally called as Factor B, plays a role in the alternative pathway like the role of C2 in the classical pathway. Factor B binds to C3b and is activated to form proteolytic enzyme that cleaves C3. Recently, it has been reviewed systematically in the literature about the COVID-19 associated thrombosis and over activation of complement cascade^56^. A preprint by Gao et al.^62^ reported that the SARS-CoV, MERS-CoV and SARS-CoV-2 nucleocapsid (N) proteins were found to bind to MBL-associated serine protease-2 via lectin pathway of complement activation, resulting in aberrant complement activation and aggravated inflammatory lung injury^63^.

C-reactive protein (CRP) is a normal plasma protein and elevates during cytokine-mediated response to most forms of tissue injury, infection and inflammation and serum CRP values are widely measured in clinical practice as an objective index of disease activity^64^. The upregulation of C reactive protein (CRP) that has been reported in COVID-19 patients might be an indication of excessive inflammatory stress and contribute to severe illness or even death^65–67^. Moreover, it has been shown that elevated CRP levels in COVID-19 patients is strongly associated with Venous thromboembolism, acute kidney injury, critical illness, and mortality^68^.

Type 1 interferon production is impaired in severe COVID-19 patients and leads to Acute Respiratory Distress Syndrome (ARDS) and coagulopathy. Matsuyama et al. reviewed COVID-19 pathophysiology with respective to NSP1 and ORF6 proteins via induction of signal transducer activator of transcription 1 (STAT1) dysfunction and compensatory hyper activation of STAT3^69^. IFN signaling was inhibited by upregulated EGFR and activated STAT3^70^. This review also emphasized the “STAT3 and Coagulopathy” with the production Tissue Factor induced by CRP which may have activated by STAT3 and prime the initial phase of coagulation.

Catenin beta-1 is also known as β-catenin. Activation of β-catenin, the primary mediator of the ubiquitous Wnt signaling pathway, alters the immune system in lasting and harmful ways^71^. It has been demonstrated that the activation of Wnt/ β-catenin signaling enhances influenza virus replication^72^. Wnt signaling is a complex mechanism of signal transduction pathways mediated by multiple signaling molecules. These molecules are involved in many disease conditions^73^. Specifically, Wnt family genes FZD4, FZD5, CTNNB1 and downstream targets CCDN1, VEGFA, axin2 were upregulated in end-stage of Pulmonary Arterial Hypertension condition, which is a life-threatening disease associated with increase pulmonary pressures, subsequently followed by development of right-sided heart failure^73^.

This study has limitations. Most importantly we have used a computational method to compare the conformations of human proteins with SARS-CoV-2 proteins. The underlying postulate is that shared structural homology would result in cross reactivity. We do not however have a direct computational measure of cross reactivity. Additionally, we rely on conformational similarities and do not weigh our scores for protein conformers that may be inaccessible to antibodies. Another limitation is that the available human protein structures represent approximately 35% of the human proteome. Similarly, variants of SARS-CoV-2 are concern are important, but we have kept this study focused on the wild type (Wuhan strain) SARS-CoV-2 for 2 reasons: (1) Essentially there have been no major changes in COVID-19 associated disorders/pathologies with the emergence of the new variants. (2) In the absence of reliable literature on pathologies associated with individual variants the data will be almost impossible to interpret. The final limitation of this study is that although we list 102 human genes with high structural homology to SARS-CoV-2 proteins these have not been experimentally validated. Hence, we do not claim that these are linked to human disease. Overall, the datasets generated here are “hypothesis generating” and provide a useful resource.

Evidence has emerged that SARS-CoV-2 infections are associated with auto antibodies and that these have the potential to elicit autoimmune pathologies. We have developed a novel computational approach to identify human proteins that have conformational features similar to SARS-CoV-2 proteins. Thus, there is a likelihood that these human proteins could be targeted by anti-SARS-CoV-2 antibodies. This method and list of human proteins is a resource that can be utilized to study the phenomenon of autoimmune pathologies associated with COVID-19.

## Conclusions

In this theoretical study we have identified multiple human proteins with strong structural homology to SARS-CoV-2 proteins. Of these, we posit 102 human proteins could potentially be both (i) associated with COVID-19 related clinical pathologies based on their known function and (ii) targeted by anti-SARS-CoV-2 antibodies. The data sets we have generated using novel computational methods present testable hypotheses to elucidate molecular mechanisms that could explain the complex multi-system disorders associated with COVID-19.

## Supplementary Information

Supplementary Information 1.

Supplementary Table 1.

Supplementary Table 2.

Supplementary Table 3.

Supplementary Table 4.

Supplementary Table 5.

## Data Availability

The datasets generated as a result of this experiment can be obtained from the corresponding author upon reasonable request.
