# Supplementary material for: A structural homology approach to identify potential cross-reactive antibody responses following SARS-CoV-2 infection
Source: Sci Rep. 2022 Jul 6;12:11388. doi: 10.1038/s41598-022-15225-3 (PMC9259575; doi:10.1038/s41598-022-15225-3)
Supplement: Supplementary file 1 — Supplementary Information 1. [file 41598_2022_15225_MOESM1_ESM.docx]

>compare_covid_proteime_probis.py

import glob

import multiprocessing

import subprocess

from tqdm import tqdm

import os

def extract_surface(prot_chain):

prot=prot_chain[:4]

chain=prot_chain[-1]

proc=subprocess.call('probis -extract -f1 ../pdbs/%s.pdb -c1 %s -srffile ../srf_files/%s-%s.srf >>srf_output 2>> srf_error '%(prot,chain,prot,chain),shell=True)

return(0)

def align_srf_files(covid_srf):

chain=covid_srf[15]

file_prefix=covid_srf.split('/')[1].split('.')[0]

command='probis -ncpu 24 -surfdb -sfile proteome_srf_files.txt -f1 %s -c1 %s -nosql results/%s/%s.nosql >> results/%s/%s.output 2>> results/%s/%s.error'%(covid_srf,chain,file_prefix,file_prefix,file_prefix,file_prefix,file_prefix,file_prefix)

subprocess.call(command,shell=True)

return(0)

all_pdbs=glob.glob('../pdbs/*.pdb')

human_chains=[i.strip() for i in open('human_proteome_chains_list.txt').readlines()]

covid_chains=[i.strip() for i in open('covid_chains_list.txt').readlines()]

human_dict={}

covid_dict={}

for i in human_chains:

if i[:4] in human_dict:

human_dict[i[:4]].append(i[-1])

else:

human_dict[i[:4]]=[i[-1]]

for i in covid_chains:

if i[:4] in covid_dict:

covid_dict[i[:4]].append(i[-1])

else:

covid_dict[i[:4]]=[i[-1]]

all_chains=human_chains+covid_chains

# Extract the surface files from all chains This only needs to be done once. Results are saved in the srf_files directory

'''

with multiprocessing.Pool(23) as pool:

results=list(tqdm(pool.imap(extract_surface, all_chains),total=len(all_chains)))

pool.close()

pool.join()

'''

srf_files=glob.glob('../srf_files/*.srf')

covid_srf_files=[]

## Making sure that the srf files exist

#Some proteome files did not but these are all here

for covid_chain in covid_chains:

srf_name='srf_files/%s-%s.srf'%(covid_chain[:4],covid_chain[-1])

if srf_name in srf_files:

covid_srf_files.append(srf_name)

else:

print('Error')

# make a srf file listing for the human proteome

# not all pdbs are proteins

'''

human_srf_files=[]

outfile=open('proteome_srf_files.txt','w')

for human_chain in human_chains:

srf_name='srf_files/%s-%s.srf'%(human_chain[:4],human_chain[-1])

if srf_name in srf_files:

outfile.write('%s %s\n'%(srf_name,human_chain[-1]))

else:

print('Error')

outfile.close()

'''

for i in tqdm(covid_srf_files):

try:

os.mkdir('results/%s'%i.split('/')[1].split('.')[0])

except OSError as error:

pass

align_srf_files(i)

>download_pdbs.py

import glob

import subprocess

import multiprocessing

import tqdm

pool=multiprocessing.Pool(23)

pdbs_have=glob.glob('*.pdb.gz')

pdbs_have=[i.split('.')[0] for i in pdbs_have]

infile = [i.split('\t')[0].strip() for i in open("sars_pdb_ids.txt").readlines() ]

infile=list(set([i for i in infile if i not in pdbs_have]))

x=len(infile)

def dl_file(i):

subprocess.call('wget https://files.rcsb.org/download/%s.pdb.gz'%i,shell=True)

for i in infile:

dl_file(i)

#for _ in tqdm.tqdm(pool.imap_unordered(dl_file, infile), total=len(infile)):

# pass

>extract_json_from_nosql.py

import glob

import subprocess

from tqdm import tqdm

nosql_files=glob.glob('../results/*/*.nosql')

print(nosql_files[0])

for nsfile in tqdm(nosql_files):

prefix=nsfile.split('/')[2]

pdbId,chain=prefix.split('-')

bash_command='~/bioinformatics_tools/probis -results -f1 ../pdbs/%s.pdb -c1 %s -out ../results/%s/ -nosql %s -json %s.json -z_score 2'%(pdbId,chain,prefix,nsfile,prefix)

subprocess.call(bash_command,shell=True)

>filter_human_pdbs.py

import glob

import re

import subprocess

pdb_list=glob.glob('*.pdb')

print(pdb_list[:10])

pattern_id=re.compile('^SOURCE\s+[0-9]+\s+ORGANISM_TAXID:\s([0-9]+);')

pattern_description=0

ct=1

ct2=0

tot=len(pdb_list)

for pdb in pdb_list:

ids=[]

lines=open(pdb).readlines()

for l in lines:

ans=(re.match(pattern_id,l))

if ans:

ids.append(ans.groups()[0])

#print(set(ids))

if set(ids)!={'9606'} :

print('%d of %d %s %s'%(ct,tot,','.join(set(ids)),pdb))

ct2+=1

else:

subprocess.call('cp %s ../all_human_chain/'%pdb,shell=True)

ct+=1

print(ct2)

>parse_and_filter_json_files.py

import json

import glob

from tqdm import tqdm

json_files=glob.glob('../results/*/*.json')

json_files=[i for i in json_files if i.split('/')[-1].split('.')[0] not in ['info','query']]

print(json_files)

outfile=open('probis_results_parsed.csv','w')

outfile.write('covid_pdb,covid_chain,human_pdb,human_chain,alignment_number,aligned_vertices,e_value,rmsd,sva,z_score,alignment_score\n')

for i in tqdm(json_files):

print(i)

covid_pdb,covid_chain=i.split('/')[2].split('-')

infile=json.load(open(i))

for entry in infile:

human_pdb=entry['pdb_id']

human_chain=entry['chain_id']

for alignment in entry['alignment']:

alNum=alignment['scores']['alignment_no']

alVert=alignment['scores']['aligned_vertices']

eVal=alignment['scores']['e_value']

rmsd=alignment['scores']['rmsd']

sva=alignment['scores']['sva']

zScore=alignment['scores']['z_score']

alScore=alignment['scores']['alignment_score']

outfile.write('%s,%s,%s,%s,%d,%d,%f,%f,%f,%f,%f\n'%(covid_pdb,covid_chain,human_pdb,human_chain,alNum,alVert,eVal,rmsd,sva,zScore,alScore))

outfile.close()
