# Supplementary material for: A structural homology approach to identify potential cross-reactive antibody responses following SARS-CoV-2 infection
Source: Sci Rep. 2022 Jul 6;12:11388. doi: 10.1038/s41598-022-15225-3 (PMC9259575; doi:10.1038/s41598-022-15225-3)
Supplement: Supplementary file 4 — Supplementary Table 3. [file 41598_2022_15225_MOESM4_ESM.rtf]

Supplementary Table 3: A list of all databases and tools used in the study along will the URLs.
Database/Software	URL	Usage	
SwissProt	https://www.uniprot.org/	used to identify proteins in the proteome	
RCSB PDB Bank	https://www.rcsb.org/	Used to retrieve relevant human proteome and Covid19 pdb files	
ProBis Algorithm	http://insilab.org/probis-algorithm/	Used for structural homology comparisons between human and Covid19 protein chains	
PyMol	https://pymol.org	Used for graphical representation of protein structures	
